# Supplementary material for: Health and cost impact of stepping down asthma medication for UK patients, 2001–2017: A population-based observational study
Source: PLoS Med. 2020 Jul 21;17(7):e1003145. doi: 10.1371/journal.pmed.1003145 (PMC7373267; doi:10.1371/journal.pmed.1003145)
Supplement: S1 Study Protocol — (DOC) [file pmed.1003145.s010.doc]

# ISAC APPLICATION FORM

# PROTOCOLS FOR RESEARCH USING THE CLINICAL PRACTICE RESEARCH DATALINK (CPRD)

| For ISAC use only | | |
| --- | --- | --- |
| Protocol No.  Submission date  (DD/MM/YYYY) | ...........................  ........................... | ***IMPORTANT***  *Please refer to the* ***guidance*** *for ‘****Completing the ISAC application form’*** *found on the CPRD website (*[*www.cprd.com/isac*](http://www.cprd.com/isac)*). If you have any queries, please contact the ISAC Secretariat at* [*isac@cprd.com*](mailto:isac@cprd.com)*.* |

| SECTION A: GENERAL INFORMATION ABOUT THE PROPOSED RESEARCH STUDY | | |
| --- | --- | --- |
| 1. Study Title**^§^** (*Please state the study title below)*   Effect of stepping down (reducing) a stable asthma patient’s maintenance medication on the risk of an acute asthma attack  *^§^Please note: This information will be published on the CPRD’s website as part of its transparency policy.* | | |
| 1. **Has any part of this research proposal or a related proposal been previously submitted to ISAC?**   Yes **^*^** No ✓  **If yes, please provide the previous protocol number/s below. Please also state in your current submission how this/these are related or relevant to this study.* | | |
| 1. **Has this protocol been peer reviewed by another Committee? (e.g. grant award or ethics committee)**   Yes**^*^** No ✓  **If Yes, please state the name of the reviewing Committee(s) below and provide an outline of the review process and outcome as an Appendix to this protocol :* | | |
| 1. **Type of Study** (please tick all the relevant boxes which apply)  | Adverse Drug Reaction/Drug Safety |  | Drug Effectiveness |  | | --- | --- | --- | --- | | Drug Utilisation | **✓** | Pharmacoeconomics |  | | Disease Epidemiology | **✓** | Post-authorisation Safety |  | | Health care resource utilisation | **✓** | Methodological Research |  | | Health/Public Health Services Research |  | Other^*^ |  |     *^*^If Other, please specify the type of study in the lay summary* | | |
| 1. **Health Outcomes to be Measured^§^**   **^§^***Please note:* *This information will be published on CPRD’s website as part*  *of its transparency policy.*  Please summarise below the primary/secondary health outcomes to be measured in this research protocol:   | - Asthma attacks |  |  | | --- | --- | --- | | - Reducing asthma medication |  |  | | - Increasing asthma medication |  |  |   [Please add more bullet points as necessary] | | |
| 1. **Publication: This study is intended for** (please tick all the relevant boxes which apply)**:**   Publication in peer-reviewed journals **✓** Presentation at scientific conference **✓**  Presentation at company/institutional meetings **✓** Regulatory purposes  Other**^*^**  **If Other, please provide further information:* | | |
| SECTION B: INFORMATION ON INVESTIGATORS AND COLLABORATORS | | |
| 1. **Chief Investigator^§^**   Please state the full name, job title, organisation name & e-mail address for correspondence - see guidance notes for eligibility. Please note that there can only be one Chief Investigator per protocol.  Dr Jennifer Quint  Senior Clinical Lecturer in Respiratory Epidemiology  Imperial College London  j.quint@imperial.ac.uk  **^§^***Please note:* *The name and organisation of the Chief Investigator and will be published on CPRD’s website as part of its transparency policy*  CV has been previously submitted to ISAC ✓ **CV number:**  042_15CEPSL  A new CV is being submitted with this protocol  An updated CV is being submitted with this protocol | | |
| 1. **Affiliation of Chief Investigator** (full address)   Respiratory Epidemiology, Occupational Medicine and Public Health  G48, Emmanuel Kaye Building,  Manresa Road  National Heart and Lung Institute  Imperial College  London, SW3 6LR  Tel +44 (0) 207 594 8824 | | |
| 1. **Corresponding Applicant^§^**   Please state the full name, affiliation(s) and e-mail address below:  Dr Chloe Bloom (address and affliliations as above)  chloe.bloom06@imperial.ac.uk  **^§^***Please note:* *The name and organisation of the corresponding applicant and their organisation name will be published on CPRD’s website as part of its transparency policy*  Same as chief investigator  CV has been previously submitted to ISAC ✓ **CV number:** 312_16  A new CV is being submitted with this protocol  An updated CV is being submitted with this protocol | | |
| 1. **List of all investigators/collaborators^§^**   Please list the full name, affiliation(s) and e-mail address* of all collaborators, other than the Chief Investigator below:  **^§^***Please note: The name of all investigators and their organisations/institutions will be published on CPRD’s website as part of its transparency policy*  Other investigator:  CV has been previously submitted to ISAC  **CV number :**  A new CV is being submitted with this protocol  An updated CV is being submitted with this protocol  Other investigator:  CV has been previously submitted to ISAC  **CV number:**  A new CV is being submitted with this protocol  An updated CV is being submitted with this protocol  Other investigator:  CV has been previously submitted to ISAC  **CV number:**  A new CV is being submitted with this protocol  An updated CV is being submitted with this protocol  Other investigator:  CV has been previously submitted to ISAC  **CV number:**  A new CV is being submitted with this protocol  An updated CV is being submitted with this protocol  [Please add more investigators as necessary]  **Please note that your ISAC application form and protocol* ***must*** *be copied to all e-mail addresses listed above at the time of submission of your application to the ISAC mailbox. Failure to do so will result in delays in the processing of your application.* | | |
| 1. **Conflict of interest statement***   Please provide a draft of the conflict (or competing) of interest (COI) statement that you intend to include in any publication which might result from this work  Dr Quint’s research group has received funding from the MRC, Welcome Trust, BLF, Insmed, Bayer, GSK, the Health Foundation, BI and AZ for other projects, none of which relate to this work.  **Please refer to the International Committee of Medical Journal Editors (ICMJE) for guidance on what constitutes a COI.* | | |
| 1. **Experience/expertise available**   Please complete the following questions to indicate the experience/ expertise available within the team of investigators/collaborators actively involved in the proposed research, including the analysis of data and interpretation of results.  **Previous GPRD/CPRD Studies** **Publications using GPRD/CPRD data**  None  1-3  > 3 ✓ ✓ | | |
| **Experience/Expertise available** | **Yes** | **No** |
| **Is statistical expertise available within the research team?**  *If yes, please indicate the name(s) of the relevant investigator(s)*  Bloom, Quint | ✓ |  |
| **Is experience of handling large data sets (>1 million records) available within the research team?**  *If yes, please indicate the name(s) of the relevant investigator(s)*  Bloom, Quint | ✓ |  |
| **Is experience of practising in UK primary care available to or within the research team?**  *If yes, please indicate the name(s) of the relevant investigator(s)*  Bloom, Quint | ✓ |  |
| 1. **References relating to your study**   Please list up to 3 references (most relevant) relating to your proposed study:   1. British Thoracic Society. BTS/SIGN British guideline on the management of asthma. (2016). Available at: <https://www.brit-thoracic.org.uk/standards-of-care/guidelines/btssign-british-guideline-on-the-management-of-asthma/>. 2. Ahmad, S., Kew, K. M. & Normansell, R. Stopping long-acting beta _2_ -agonists (LABA) for adults with asthma well controlled by LABA and inhaled corticosteroids. *Cochrane Database Syst. Rev.* CD011306 (2015). doi:10.1002/14651858.CD011306. 3. Crossingham, I., Evans, D. J., Halcovitch, N. R. & Marsden, P. A. Stepping down the dose of inhaled corticosteroids for adults with asthma. *Cochrane Database Syst. Rev.* **2,** CD011802 (2017) | | |
| SECTION C: ACCESS TO THE DATA | | |
| 1. **Financial Sponsor of study^§^**   **^§^***Please note:* *The name of the source of funding will be published on CPRD’s website as part of its transparency policy*  Pharmaceutical Industry  *Please specify name and country:*  Academia  *Please specify name and country:*Imperial College London  Government / NHS  *Please specify name and country:*  Charity *Please specify name and country:*  Other  *Please specify name and country:*  None | | |
| 1. **Type of Institution conducting the research**   Pharmaceutical Industry *Please specify name and country:*  Academia **✓** *Please specify name and country:* Imperial College, UK  Government Department *Please specify name and country:*  Research Service Provider *Please specify name and country:*  NHS *Please specify name and country:*  Other  *Please specify name and country:* | | |
| 1. **Data access arrangements**   The financial sponsor/ collaborator* has a licence for CPRD GOLD and will extract the data  The institution carrying out the analysis has a licence for CPRD GOLD and will extract the data**  A data set will be provided by the CPRD^¥€^  CPRD has been commissioned to extract the data and perform the analyses^€^  Other:  *If Other, please specify:*  **Collaborators supplying data for this study must be named on the protocol as co-applicants.*  ***If data sources other than CPRD GOLD are required, these will be supplied by CPRD*  ^¥^*Please note that datasets provided by CPRD are limited in size; applicants should contact CPRD (*[*kc@cprd.com*](mailto:kc@cprd.com)*) if a dataset of >300,000 patients is required.*  ^€^*Investigators must discuss their request with a member of the CPRD Research team before submitting an ISAC application. Please contact the CPRD Research Team on +44 (20) 3080 6383 or email (*[*kc@cprd.com*](mailto:kc@cprd.com)*) to discuss your requirements. Please also state the name of CPRD Research team with whom you have discussed this request (provide the date of discussion and any relevant reference information):*  Name of CPRD Researcher       Reference number (where available)       Date of contact | | |
| 1. **Primary care data**   Please specify which primary care data set(s) are required)  Vision only (Default for CPRD studies **✓** Both Vision and EMIS®*  EMIS® only*    *Note: Vision and EMIS are different practice management systems. CPRD has traditionally collected data from Vision practice. Data collected from EMIS is currently under evaluation prior to wider release.*  **Investigators requiring the use of EMIS data* ***must*** *discuss the study with a member of the CPRD Research team before submitting an ISAC application*  Please state the name of the CPRD Researcher with whom you have discussed your request for EMIS data:  Name of CPRD Researcher       Reference number (where available)       Date of contact | | |
| SECTION D: INFORMATION ON DATA LINKAGES | | |
| 1. **Does this protocol seek access to linked data**   Yes* ✓ No If No, please move to section E.  **Research groups which have not previously accessed CPRD linked data resources* ***must*** *discuss access to these resources with a member of the CPRD Research team, before submitting an ISAC application. Investigators requiring access to HES Accident and Emergency data, HES Diagnostic Imaging Dataset and PROMS data* ***must*** *also discuss this with a member of the CPRD Research team before submitting an ISAC application. Please contact the CPRD Research Team on +44 (20) 3080 6383 or email* [*kc@cprd.com*](mailto:kc@cprd.com) *to discuss your requirements* ***before*** *submitting your application.*  Please state the name of the CPRD Researcher with whom you have discussed your linkage request.  As our research group has extensive experience in using linked data, contact with a CPRD researcher is not required.  Name of CPRD Researcher       Reference number (where available)       Date of contact  *Please note that as part of the ISAC review of linkages, your protocol may be shared - in confidence - with a representative of the requested linked data set(s) and summary details may be shared - in confidence - with the Confidentiality Advisory Group of the Health Research Authority.* | | |
| 1. **Please select the source(s) of linked data being requested^§^**   *^§^Please note: This information will be published on the CPRD’s website as part of its transparency policy.*   | ONS Death Registration Data | MINAP (Myocardial Ischaemia National Audit Project) | | --- | --- | | HES Admitted Patient Care | Cancer Registration Data* | | HES Outpatient | PROMS (Patient Reported Outcomes Measure)** | | HES Accident and Emergency | CPRD Mother Baby Link | | HES Diagnostic Imaging Dataset |  |     Practice Level Index of Multiple Deprivation (Standard)  Practice Level Index of Multiple Deprivation (Bespoke)  Patient Level Index of Multiple Deprivation***  Patient Level Townsend Score ***  Other**** *Please specify:*  **Applicants seeking access to cancer registration data must complete a Cancer Dataset Agreement form (available from CPRD). This should be submitted to the ISAC as an appendix to your protocol.* *Please also note that applicants seeking access to cancer registry data must provide consent for publication of their study title and study institution on the UK Cancer Registry website.*  ***Assessment of the quality of care delivered to NHS patients in England undergoing four procedures: hip replacement, knee replacement, groin hernia and varicose veins. Please note that patient level PROMS data are only accessible by academics*  **** ‘Patient level IMD and Townsend scores will not be supplied for the same study*  *****If “Other” is specified, please provide the name of the individual in the CPRD Research team with whom this linkage has been discussed.*  Name of CPRD Researcher       Reference number (where available)       Date of contact | | |
| 1. **Total number of linked datasets requested including CPRD GOLD**   Number of linked datasets requested *(practice/ ’patient’ level Index of Multiple Deprivation, Townsend Score or the CPRD Mother Baby Link should* ***not*** *be included in this count)*  3  *Please note: Where ≥5 linked datasets are requested, approval may be required from the Confidentiality Advisory Group (CAG) to access these data* | | |
| 1. **Is linkage to a local^¥^ dataset with <1 million patients being requested?**   Yes *  No ✓  **If yes, please provide further details:*  **^¥^** *Data from defined geographical areas i.e. non-national datasets.* | | |
| 1. **If you have requested one or more linked data sets, please indicate whether the Chief Investigator or any of the collaborators listed in question 5 above, have access to these data in a patient identifiable form (e.g. full date of birth, NHS number, patient post code), or associated with an identifiable patient index.**   Yes*  No ✓  ** If yes, please provide further details:* | | |
| 1. **Does this study involve linking to patient *identifiable* data (e.g. hold date of birth, NHS number, patient post code) from other sources?**   Yes  No ✓ | | |
| SECTION E: VALIDATION/VERIFICATION | | |
| 1. **Does this protocol describe a purely observational study using CPRD data?**   Yes* ✓ No**  ** Yes: If you will be using data obtained from the CPRD Group, this study does not require separate ethics approval from an NHS Research Ethics Committee.*  *** No: You may need to seek separate ethics approval from an NHS Research Ethics Committee for this study. The ISAC will provide advice on whether this may be needed.* | | |
| 1. **Does this protocol involve requesting any additional information from GPs?**   Yes*  No ✓  * *If yes, please indicate what will be required:*  Completion of questionnaires by the GP*^ψ^* Yes  No  Is the questionnaire a validated instrument? Yes  No  If yes, has permission been obtained to use the instrument? Yes  No  Please provide further information:  Other (please describe)  *^ψ^ Any questionnaire for completion by GPs or other health care professional must be approved by ISAC before circulation for completion.* | | |
| 1. **Does this study require contact with patients in order for them to complete a questionnaire?**   Yes*  No ✓  **Please note that any questionnaire for completion by patients must be approved by ISAC before circulation for completion.* | | |
| 1. **Does this study require contact with patients in order to collect a sample?**   Yes*  No ✓  ** Please state what will be collected:* | | |
| SECTION F: DECLARATION | | |
| 1. **Signature from the Chief Investigator**  - I have read the guidance on ‘***Completion of the ISAC application form****’* and ‘***Contents of CPRD ISAC Research Protocols***’ and have understood these; - I have read the submitted version of this research protocol, including all supporting documents, and confirm that these are accurate. - I am suitably qualified and experienced to perform and/or supervise the research study proposed. - I agree to conduct or supervise the study described in accordance with the relevant, current protocol - I agree to abide by all ethical, legal and scientific guidelines that relate to access and use of CPRD data for research - I understand that the details provided in sections marked with (^§^) in the application form and protocol will be published on the CPRD website in line with CPRD’s transparency policy. - I agree to inform the CPRD of the final outcome of the research study: publication, prolonged delay, completion or termination of the study.   Name: Jennifer Quint Date: 10/4/2017 e-Signature (type name): JKQuint | | |

**PROTOCOL INFORMATION REQUIRED**

The following sections below **must** be included in the CPRD ISAC research protocol. Please refer to the guidance on ‘***Contents of CPRD ISAC Research Protocols***’ ([www.cprd.com/isac](http://www.cprd.com/isac)) for more information on how to complete the sections below. Pages should be numbered. All abbreviations must be defined on first use.

| **Applicants must complete all sections listed below**  **Sections which do not apply should be completed as ‘*Not Applicable’*** |
| --- |
| 1. **Study Title^§^**   **^§^***Please note:* *This information will be published on CPRD’s website as part of its transparency policy*  Effect of stepping down (reducing) a stable asthma patient’s maintenance medication on the risk of an acute asthma attack |
| 1. **Lay Summary (Max. 200 words)^§^**   **^§^***Please note:* *This information will be published on CPRD’s website as part of its transparency policy*  Asthma is the most common chronic lung disorder in the UK; approximately 1 in 9 people have received an asthma diagnosis. The mainstay of treatment is regular inhaled medication, called ‘maintenance medication’, which aims to prevent daily asthma symptoms (including breathlessness, wheeze and cough) and asthma attacks (sudden worsening of symptoms requiring urgent treatment). If an asthma patient does not have many symptoms, and only infrequently uses their asthma ‘reliever medication’ (gives immediate symptom relief), the patient is described as having ‘well-controlled asthma’. Once a patient is well controlled, i.e. has stable asthma, guidelines recommend considering reducing their maintenance medication to reduce the risk of side effects; for example, all maintenance medications include inhaled corticosteroids (side effects include voice hoarseness, cataracts or slowed growth in children). However, recent nationally-recognised medical reviews have concluded there is insufficient evidence from trials as to whether stepping down (reducing) asthma treatment is beneficial or not. Our study aims to, firstly, determine how common stepping down (reducing) asthma treatment is in UK clinical practice, and secondly, estimate the effect of this clinical practice on asthma attacks, and asthma control, in the real-world. |
| 1. **Technical Summary (Max. 200 words**)**^§^**   **^§^***Please note:* *This information will be published on CPRD’s website as part of its transparency policy*  We will used CPRD to identify asthma patients and their medication, and describe how often patients step down, or step up, their maintenance asthma medication, according to British asthma clinical guidelines, between 2000 and 2016. We will use a Poisson regression model to identify factors associated with stepping down, and stepping up, asthma maintenance medication.  Next we will use CPRD, linked with HES and ONS, to conduct a matched cohort study, the exposures will be stepping down asthma treatment, according to clinical guidelines, and the main outcome will be asthma attacks (treated in primary and secondary care). A secondary outcome will be stepping asthma treatment back up. Patients will be matched by GP practice, asthma severity, age and gender. A Cox proportional hazards model will be used to estimate the hazard ratios and their corresponding 95% confidence intervals. Multiple potential confounders or effect modifiers will be included in the model to obtain adjusted estimates, and to identify factors affecting the association between the exposure and the outcomes. |
| 1. **Objectives, Specific Aims and Rationale**   Main objective  Measure the frequency of stepping down asthma treatment in stable patients and the subsequent impact on patient’s health.  Specific aims and rationale:   1. Measure the extent and temporal pattern of stepping down (decreasing) or stepping up (increasing) asthma patient’s maintenance medication, between 2000 and 2017, in the UK. 2. Identify factors that are associated with being stepped down, compared to staying on the same medication, in a stable asthma population (cohort 1).   Rationale: it is currently not known how often patients have their asthma medication changed in the UK or the factors that are associated with the probability of a stable patient being stepped down.   1. Measure the relative rate of an asthma attack (primary outcome) in a stable population that had been stepped down compared to a matched (age, gender, disease severity and GP practice) stable asthma population that had not been stepped down (cohort 2). 2. Identify factors that are associated with (confounders or effect modifiers) the risk of an asthma attack, after being stepped down (cohort 2). 3. Measure the relative rate of stepping back up an asthma patient’s maintenance medication (secondary outcome), in the asthma stable population that had been stepped down, compared to a matched (age, gender and maintenance medication) stable asthma population that had not been stepped down (cohort 2). 4. Identify factors that are associated with (confounders or effect modifiers) being stepped back up, after being stepped down (cohort 2).   Rationale: the effect of stepping down medication in a real-world population has not been well estimated. There are two major consequences to consider, firstly an increased risk of an asthma attack, and secondly worsening of asthma control; as a proxy we will use stepping the medication back up as asthma control scores are not well recorded in CPRD and the use of respiratory symptoms alone could lead to misclassification. |
| 1. **Study Background**   The British Thoracic Society (BTS) asthma management guidelines^1^, are very well adhered to clinical guidelines by healthcare workers in both primary and secondary care. These guidelines also form the basis for the British National Formulary asthma management pathway. The management of asthma is through regular use of inhaled medication. This comes in two forms, reliever medication for immediate symptom relief, and maintenance medication to prevent asthma attacks and keep a patient’s day to day asthma symptoms under good control. The BTS guidelines recommend using maintenance medication in a stepwise approach, starting with low doses of inhaled corticosteroids (ICS), and increasing with high ICS doses and additional inhaled and oral medications, according to symptoms (breathlessness, wheeze and cough).  The BTS asthma guidelines and international asthma guidelines (European and American^2,3^) suggest that when asthma is controlled for at least 3 months, medication should be stepped down (reduced). This is to reduce the risk of adverse effects from the medication. Although the adverse effects are uncommon, especially at low doses, concerns still remain; particularly at high doses of ICS, where local side effects can include hoarseness and oral candidiasis, and systemic effects can include osteoporosis, cataracts, adrenal axis suppression as well as potential increased pneumonia risk^4^. In children there is an additional concern of reduced growth secondary to ICS^5^. Another commonly used asthma maintenance medication is long-acting beta agonists (LABA), where there still remains some debate about their safety (evidence from randomised controlled trials, RCTs, is still inconclusive^6^). In addition, asthma medications are expensive and unnecessary treatment costs are best avoided in the current economic climate.  Cochrane systematic reviews have recently assessed multiple randomised controlled trials looking at (1) RCTs that have stopped LABA in well controlled adults and children^7,8^ and (2) RCTS that have stepped down ICS in well controlled asthma patients^9^. None of these reviews, or more recent trials^10^, found conclusive evidence for or against stepping down asthma patient’s medication. Furthermore, in such trials it must be remembered that inhaler technique is likely to have been optimised and adherence encouraged, unlike true clinical practice, and patients may not have represented the general asthma population as seen in primary care in the UK. Stepping down medication in stable asthma patients is thought not to occur frequently. A real-world study of asthma patients in the United States, using claims-based data, seemed to suggest that asthma attacks occur frequently in patients that are stepped down, but they did not have a comparison group that had not been stepped down^11^. Therefore, we wish to determine how common stepping down maintenance asthma treatment is in the UK, and the health impact of this clinical practice when it has occurred. |
| 1. **Study Type**   Hypothesis testing |
| 1. **Study Design**   Historical matched cohorts:  (A) **cohort 1**: to determine factors associated with stepping down (unmatched)  (B) **cohort 2:** to determine the effect of stepping down (matched) |
| 1. **Feasibility counts**   From our previous study (protocol 17_090R, submitted for publication), we have shown that between 2000 and 2016 there were between 200,000 and 250,000 asthma patients on regular inhalers every year in CPRD. Of these, approximately 2% had their inhalers switched annually for clinical reason; the predominant reason (over 95%) was due to a change in medication dose. This would amount to approximately 4,500 patients annually, but assuming only one-third is stepped down, this would give a feasibility count of around 1,350 exposed patients for the matched cohort every year. |
| 1. **Sample size considerations**   Cohort 1: the sample size of 569,901 asthma patients (protocol 17_090R) between 2000 and 2016 will be more than sufficient for the Poisson model regression analysis.  Cohort 2: making the following assumptions for the Cox proportional hazards model (matched cohort), based on previous studies as described in the background: 90% power, alpha 0.05, 35% exacerbate in the exposed group, 20% exacerbate in the unexposed group, and correlation coefficient of 0.2 for failure between paired subjects, we would require 2,148 exposed patients. |
| 1. **Data Linkage Required (if applicable):^§^**   **^§^***Please note that the data linkage/s requested in research protocols will be published by the CPRD as part of its transparency policy*  The main study outcome is an asthma attack but these are treated by both GP practices (primary care) and in hospitals (secondary care) – therefore it is necessary to have linked data, otherwise we would be excluding a proportion of asthma attacks.  Linkage to IMD is required to understand if socioeconomic status is associated with asthma attacks or not after stepping down as it is known to be associated with an asthma attack risk. |
| 1. **Study population**   The study population for both cohorts will be   - Asthma patients as defined by validated asthma Read codes^12^ - Patients who have at least one year of research acceptable data - All ages - Patients will be eligible to enter the study period after the latest of: 1 Jan 2000, continuous CPRD registration, index date of their asthma diagnosis and their up to standard registration date, 12-months of maintenance treatment. - Follow-up will end at the first date of: end of the study observation period, death, transfer out date or last collection date from the practice. Additionally in cohort 1 patients will be censored if they step up their treatment. - For cohort 2, to ensure patients are ‘stable’, according to guidelines (which state a stable patient after 3 months can be considered for stepping down), they must have (i) been on the same consecutive prescription drug class and dose for at least 3 prescriptions, each within 3 months of each other but more than 20 days of each other (this is to ensure patients are stable on their medication), (ii) have had no asthma attack for at least 3 months (sensitivity analyses will use more stringent cut-offs for ‘stable’ asthma of 6 months and 12 months, for both criteria (i) and (ii)) |
| 1. **Selection of comparison group(s) or controls**   In cohort 1, the analysis will involve comparing those exposed or unexposed for all measured covariates  In cohort 2, the matched group will be patients whose maintenance medication had not changed at the point of matching during study follow-up. |
| 1. **Exposures, Health Outcomes^§^ and Covariates**   **^§^***Please note:* *Summary information on health outcomes (as included on the ISAC application form above )will be published on CPRD’s website as part of its transparency policy*  Exposure (cohort 2) or Outcome (cohort 1)   - The main exposure in cohort 2, or outcome in cohort 1, will be stepping down asthma medication - This will be defined as the fourth consecutive prescription that is a step down as per the BTS stepwise approach - The following will be defined as ‘stepping down’ - (a) Same ICS drug but at a lower dose equivalent to one of the BTS categories (very low, low, medium or high), these are defined clearly in the guidelines and depend on the age of the patient   (b) Removal of LABA, from previous treatment with both LABA & ICS, while maintaining the same ICS dose  (c) Removal of leukotriene receptor antagonist if the child is under 5 years old   - The following will not be included as ‘stepping down’: drop of ICS within one of the BTS categories, or removal of LABA and ICS, or removal of other asthma medications, as these are not according to BTS guidelines   Outcome (cohort 2)   - Asthma attack as defined by a short course of oral corticosteroids prescribed by GP practice (not on day of an annual review) or hospitalisation with asthma attack (HES) or death from asthma (ONS data). - As a proxy for a short course of oral corticosteroids, where data is missing, <300mg oral corticosteroids will be used^13^ - A secondary outcome of stepping back up asthma medication, this will be a proxy for worsening asthma control, which is difficult to measure in CPRD due to high amount of missing data for the asthma control questionnaires (respiratory symptoms are too non-specific and can be chronic or acute, which is crucial here). Stepping up will be defined as stepping back up to the next level in the BTS stepwise approach.   Covariates (and exposures in cohort 1)   - Gender - Age (categorical by 0-5 years, 5-12 years, 12-18 years, 18-55 years, 55+ years) - Socioeconomic status (IMD) - Body mass index - Smoking history - Asthma severity (BTS step is used in the UK to define asthma severity and will be measure by medication use 12-months prior to study start^13^) - Short acting bronchodilator agonist use (asthma reliever) will be measured as an average number of canisters per year and divided into 4 categories - Co-morbidities: atopy, depression, anxiety, COPD co-diagnosis (using validated defintion^14^), gastro-oesophageal reflux disease, rhinitis - Season stepped down in - Asthma management plan given - Inhaler technique checked - Annual asthma review in past 12 months |
| 1. **Data/ Statistical Analysis**   Descriptive analysis:  The frequency of stepping up or down will be measured for each year between 2000 and 2016, and for each BTS step (asthma severity level). Also, the possible number of asthma patients that could have been stepped down will be calculated if stepping down is not found to be detrimental to patient’s health.  Cohort 1:  Incidence rates of stepping down will be estimated for each covariate. Poisson regression model will then be used to estimate the incidence rate ratio for each of the covariates (crude IRR), and adjusted for other covariates (adjusted IRR), to understand the factors leading to being stepped down or remaining on the same medication.  Cohort 2:  Cox proportional hazards model will be used for time to first asthma attack after stepping down (or index time if matched unexposed patient). This analysis will be repeated to allow for repeated outcomes using Andersen-Gill methodology. The proportional hazards assumption will be tested, and we will perform analysis stratified by follow up time if this is not met. Covariates will be tested for effect modification, depending on the analysis from cohort 1, using likelihood ratio test, if found then stratified analysis will be carried out to help understand if any factors are associated with a better outcome than others. |
| 1. **Plan for addressing confounding**   We will adjust or match for the covariates listed in section M.  In addition, if there are found to be multiple confounding factors in cohort 1 (i.e. as to why patients are selected for stepping down), we will consider using propensity score methodology for the analysis of cohort 2. For example, if we used matching, logistic regression will be used to estimate the probability of being stepped down or not (exposed or not), based on the covariates, to calculate a propensity score for each patient. Patients will then be matched on age, gender, BTS step and propensity score. Every patient that is stepped down (exposed) will be matched with replacement, to each unexposed patient, on the basis of the closest propensity score and other matching variables. The distributional balance in the exposed and unexposed groups will be assessed for all covariates in the model by plotting relative density functions to look at the distribution of the scores. |
| 1. **Plans for addressing missing data**   We are not expecting missing data except for the covariates BMI and smoking history where data could be missing, but would be missing at random; therefore, we would undertake both a complete case analysis and consider using multiple imputation.  If data was found not to be missing at random, but was at least 80% complete, we would use a complete case analysis and discuss any biases that may occur as a result of adopting that approach. Where multiple imputation is not appropriate and there are large quantities of data missing, we will consider using those covariates only as part of a secondary analysis and will discuss any biases and limitations that occur as a result of that. |
| 1. **Patient or user group involvement (if applicable)**   The research question was discussed with patients in our respiratory clinics to gauge their feelings on having their asthma treatment stepped down; overwhelmingly patients spoke of concern for their asthma if they stepped down their medication, even understanding this would reduce their side-effect risk from the medication. Patients will not be involved further in the study, but we will share results with relevant patient groups. |
| 1. **Plans for disseminating and communicating study results, including the presence or absence of any restrictions on the extent and timing of publication**   We will present our findings at national and international meetings and publish the results in a peer reviewed journal and share the results with relevant patient groups. There will be no restriction on the extent and timing of publication once analysis and writing up are complete. We will not include any cells with counts less than five due to anonymity concerns. |
| 1. **Limitations of the study design, data sources, and analytic methods**   A limitation of the study design is that although we will try to correct for multiple variables (by matching and adjusting in the model) there could still be residual confounding by indication, i.e. only certain patients are chosen for stepping down by unmeasured factors.  There could be misclassification as some patients may be prescribed certain medications but do not get them dispensed or adhere to them, or GPs do not adhere to guidelines (although previous work shows this is <5%). Asthma attacks will be captured using CPRD, HES and ONS based from information from previous studies. We recognise, however, that we may miss some but anticipate that this is unlikely to bias estimates of the rate ratio, assuming missed outcomes occur randomly. Mild asthma attacks will not be picked up as these are self-treated or treated only be increasing ICS, however, patients should have their medication stepped back up if they even have a mild asthma attack so this should be accounted for in the stepping up outcome. All these limitations will be reported in published findings. |
| 1. **References**   1. British Thoracic Society. BTS/SIGN British guideline on the management of asthma. (2016). Available at: https://www.brit-thoracic.org.uk/standards-of-care/guidelines/btssign-british-guideline-on-the-management-of-asthma/.  2. NHLBI. Expert panel report 3: guidelines for the diagnosis and management of asthma. *Natl. Hear. Lung, Blood Institute, Bethesda;* (2007).  3. Global Initiative for Asthma. Global Strategy for Asthma Management and Prevention. (2017).  4. Rogers, L. & Reibman, J. Stepping down asthma treatment: how and when. *Curr. Opin. Pulm. Med.* **18,** 70–5 (2012).  5. Leung, J. S. *et al.* A systematic review of adverse drug events associated with administration of common asthma medications in children. *PLoS One* **12,** e0182738 (2017).  6. Cates, C. J., Jaeschke, R., Schmidt, S. & Ferrer, M. Regular treatment with salmeterol and inhaled steroids for chronic asthma: serious adverse events. *Cochrane database Syst. Rev.* CD006922 (2013). doi:10.1002/14651858.CD006922.pub3  7. Ahmad, S., Kew, K. M. & Normansell, R. Stopping long-acting beta _2_ -agonists (LABA) for adults with asthma well controlled by LABA and inhaled corticosteroids. *Cochrane Database Syst. Rev.* CD011306 (2015). doi:10.1002/14651858.CD011306.pub2  8. Kew, K. M., Beggs, S. & Ahmad, S. Stopping long-acting beta2-agonists (LABA) for children with asthma well controlled on LABA and inhaled corticosteroids. *Cochrane Database Syst. Rev.* CD011316 (2015). doi:10.1002/14651858.CD011316.pub2  9. Crossingham, I., Evans, D. J., Halcovitch, N. R. & Marsden, P. A. Stepping down the dose of inhaled corticosteroids for adults with asthma. *Cochrane Database Syst. Rev.* **2,** CD011802 (2017).  10. Rogers, L. *et al.* Step-Down Therapy for Asthma Well Controlled on Inhaled Corticosteroid and Long-Acting Beta-Agonist: A Randomized Clinical Trial. *J. Allergy Clin. Immunol. Pract.* **6,** 633–643.e1 (2018).  11. Rank, M. A. *et al.* Long-term Outcomes After Stepping Down Asthma Controller Medications: A Claims-Based, Time-to-Event Analysis. *Chest* **148,** 630–639 (2015).  12. Nissen, F. *et al.* Validation of asthma recording in the Clinical Practice Research Datalink (CPRD). *BMJ Open* **7,** e017474 (2017).  13. Bloom, C. I. *et al.* Exacerbation risk and characterisation of the UK’s asthma population from infants to old age. *Thorax* thoraxjnl-2017-210650 (2017). doi:10.1136/thoraxjnl-2017-210650  14. Quint, J. K. *et al.* Validation of chronic obstructive pulmonary disease recording in the Clinical Practice Research Datalink (CPRD-GOLD). *BMJ Open* **4,** e005540–e005540 (2014). |
| **Amendment – 23 September 2019**  **Objectives, Specific Aims and Rationale**  Additional aims are   - 1. Include a secondary outcome to see if patients increase their reliever inhaler use after stepping down   2. Determine if the cost of medications changes after stepping down   **Study population**  The population in the statistical analysis will no longer just be stable asthma patients but all adult asthma patients (stable and not stable disease). Patients with COPD will be excluded.  **Selection of comparison group(s) or controls**  Patients in the cohort will not be matched exposed patients (stepped down) will be compared to unmatched unexposed patients (not stepped down). Confounding will be dealt with in the model instead and as described above.  **Exposures, Health Outcomes^§^ and Covariates**  As well as exposures of stepping-down the drugs ICS, LABA and LTRA (as in the ISAC already), I will also look at stepping-down two other drugs: theophylline and long-acting muscarinic antagonists; although these are relatively small numbers they may be clinically relevant.  A secondary outcome will be added – the binary outcome of ≥1 more SABA prescription in the year before the study start date compared to the year after  **Data/Statistical analysis**  The original analysis was to do (a) matched Cox regression so that GP practice could be taken into account – to assess the exposure (stepping-down treatment) on the outcome (asthma exacerbation) and (b) matched Poisson regression – to assess the exposures (all variables in section M) on the outcome (stepping-down).  However, the matching was only needed for (b) as GP practice was only thought to affect whether a patient received the outcome (stepping down) or not. GP practice was not thought necessary for (a) where the outcome was different (exacerbations).  Matching by GP practice significantly reduces the same size. Therefore, an alternative approach to account for GP practice would be to use mixed models. Therefore, the analysis would be (a) unmatched Cox regression as above; (b) unmatched mixed models logistic regression with GP practice as the random intercept. The models will be adjusted for all the variables as in section M in the ISAC.  For the secondary outcome as this is binary logistic regression model including the variables in section M will be used.  A simple cost analysis will also be carried out for design (a). Costs of inhalers in the year before stepping down will be compared to the year after stepping down. I will use NHS indicative prices to do this. |
