# Supplementary material for: Health and cost impact of stepping down asthma medication for UK patients, 2001–2017: A population-based observational study
Source: PLoS Med. 2020 Jul 21;17(7):e1003145. doi: 10.1371/journal.pmed.1003145 (PMC7373267; doi:10.1371/journal.pmed.1003145)
Supplement: S1 CPRD medcodes — CPRD, Clinical Practice Research Datalink (PDF) [file pmed.1003145.s011.pdf]

Medcode	Readterm	Variable
131	Anxiousness	anxiety
462	Panic attack	anxiety
514	Tension - nervous	anxiety
636	Anxiety states	anxiety
655	Anxiety with depression	anxiety
791	Nervous breakdown	anxiety
962	[X]Anxiety neurosis	anxiety
1582	Nervous exhaustion	anxiety
1758	Chronic anxiety	anxiety
2509	[D]Nervousness	anxiety
3076	Agoraphobia with panic attacks	anxiety
3328	General nervous symptoms	anxiety
4069	Panic disorder	anxiety
4081	[X]Panic state	anxiety
4534	Anxiety state NOS	anxiety
4634	Recurrent anxiety	anxiety
4659	Generalised anxiety disorder	anxiety
5385	[X]Other anxiety disorders	anxiety
5902	Anxiousness - symptom	anxiety
6221	Separation anxiety disorder	anxiety
6408	[X]Panic attack	anxiety
6939	Anxiety state unspecified	anxiety
7749	[X]Mild anxiety depression	anxiety
7999	Anxiety counselling	anxiety
8205	[X]Panic disorder [episodic paroxysmal anxiety]	anxiety
8424	[X]Anxious [avoidant] personality disorder	anxiety
10344	[X]Generalized anxiety disorder	anxiety
10390	Fear of death	anxiety
10723	[D]Nervous tension	anxiety
11890	C/O - panic attack	anxiety
11913	[X]Mixed anxiety and depressive disorder	anxiety
11940	Acute panic state due to acute stress reaction	anxiety
12838	Agoraphobia without mention of panic attacks	anxiety
14890	[X]Panic disorder with agoraphobia	anxiety
16729	[X]Agoraphobia without history of panic disorder	anxiety
17687	[X]Dream anxiety disorder	anxiety
19000	O/E - panic attack	anxiety
20089	General nervous symptom NOS	anxiety
23838	[X]Anxiety disorder, unspecified	anxiety
24066	[X]Other specified anxiety disorders	anxiety
25638	[X]Anxiety NOS	anxiety
26331	O/E - fearful mood	anxiety
28167	[X]Anxiety hysteria	anxiety
28381	Alleviating anxiety	anxiety
28925	Referral for guided self-help for anxiety	anxiety
34064	[X]Phobic anxiety disorder, unspecified	anxiety
35825	[X]Anxiety reaction	anxiety
44321	[X]Other mixed anxiety disorders	anxiety
50191	[X]Anxiety state	anxiety

56924	Adjustment reaction with anxious mood	anxiety
101422	Feeling low or worried	anxiety
426	SINUS ARRHYTHMIA	arrhythmia
1268	PAROXYSMAL ATRIAL FIBRILLATION	arrhythmia
1297	PAROXYSMAL ATRIAL TACHYCARDIA	arrhythmia
1381	PAROXYSMAL TACHYCARDIA NOS	arrhythmia
1501	[D]TACHYCARDIA, UNSPECIFIED	arrhythmia
1535	CARDIAC DYSRHYTHMIA NOS	arrhythmia
1536	SUPRAVENTRICULAR TACHYCARDIA NOS	arrhythmia
1664	Atrial fibrillation	arrhythmia
1757	ATRIAL FLUTTER	arrhythmia
2212	ATRIAL FIBRILLATION AND FLUTTER	arrhythmia
3115	[D] BRADYCARDIA, UNSPECIFIED	arrhythmia
3418	PAROXYSMAL VENTRICULAR TACHYCARDIA	arrhythmia
3757	ECG: ATRIAL FIBRILLATION	arrhythmia
3849	PERSISTENT SINUS BRADYCARDIA	arrhythmia
4044	CARDIAC DYSRHYTHMIAS	arrhythmia
4374	VENTRICULAR FIBRILLATION AND FLUTTER	arrhythmia
4421	HEART BEATS IRREGULAR	arrhythmia
4772	SKIPPED BEAT	arrhythmia
4827	VENTRICULAR FIBRILLATION	arrhythmia
4924	ECG: VENTRICULAR TACHYCARDIA	arrhythmia
4940	PAROXYSMAL SUPRAVENTRICULAR TACHYCARDIA	arrhythmia
5484	VENTRICULAR FLUTTER	arrhythmia
5576	SICK SINUS SYNDROME	arrhythmia
6345	H/O: ATRIAL FIBRILLATION	arrhythmia
6503	CARDIAC ARRHYTHMIAS	arrhythmia
6771	ECG: ATRIAL FLUTTER	arrhythmia
7005	SINUS TACHYCARDIA	arrhythmia
7410	SINOATRIAL NODE DYSFUNCTION NOS	arrhythmia
7794	VENTRICULAR TACHYCARDIA	arrhythmia
7827	OTHER CARDIAC DYSRHYTHMIAS	arrhythmia
8061	[D]SINUS BRADYCARDIA	arrhythmia
8651	NODAL RHYTHM DISORDER	arrhythmia
9479	IMPLANT INTRAVENOUS PACEMAKER FOR ATRIAL FIBRILLATION	arrhythmia
9515	BIGEMINAL PULSE	arrhythmia
9563	PULSE MISSED BEATS	arrhythmia
12375	HISTORY OF SUPRAVENTRICULAR TACHYCARDIA	arrhythmia
17597	ECG: SUPRAVENTRICULAR ARRHYTHMIA	arrhythmia
18268	SEVERE SINUS BRADYCARDIA	arrhythmia
18357	ECG: PAROXYSMAL ATRIAL TACHY.	arrhythmia
18746	ATRIAL FIBRILLATION MONITORING	arrhythmia
19707	ECG: VENTRICULAR ARRHYTHMIA	arrhythmia
21955	[D]RAPID HEART BEAT	arrhythmia
22874	H/O VENTRICULAR FIBRILLATION	arrhythmia
23437	Atrial fibrillation and flutter NOS	arrhythmia
23494	WANDERING ATRIAL PACEMAKER	arrhythmia
23647	PAROXYSMAL ATRIOVENTRICULAR TACHYCARDIA	arrhythmia
25266	PAROXYSMAL TACHYCARDIA UNSPECIFIED	arrhythmia
25583	CARDIAC ARREST-VENTRICULAR FIBRILLATION	arrhythmia

27463	PULSUS ALTERNANS	arrhythmia
28994	ATRIAL FIBRILLATION RESOLVED	arrhythmia
29371	ECG: VENTRICULAR ARRHYTHMIA NOS	arrhythmia
29491	PAROXYSMAL NODAL TACHYCARDIA	arrhythmia
30712	HISTORY OF VENTRICULAR TACHYCARDIA	arrhythmia
31133	OTHER CARDIAC DYSRHYTHMIA NOS	arrhythmia
31286	ECG: VENTRICULAR FIBRILLATION	arrhythmia
31690	RE-ENTRY VENTRICULAR ARRHYTHMIA	arrhythmia
35124	PAROXYSMAL SUPRAVENTRICULAR TACHYCARDIA NOS	arrhythmia
35127	NON-RHEUMATIC ATRIAL FIBRILLATION	arrhythmia
39113	[D] OTHER AND UNSPECIFIED ABNORMALITIES OF HEART BEAT	arrhythmia
41916	VENTRICULAR FIBRILLATION AND FLUTTER NOS	arrhythmia
43860	IMPLANTATION OF INTRAVENOUS ATRIAL OVERDRIVE PACEMAKEI	arrhythmia
45773	ATRIAL FIBRILLATION ANNUAL REVIEW	arrhythmia
51845	PAROXYSMAL JUNCTIONAL TACHYCARDIA	arrhythmia
52663	ECG: SUPRAVENTRIC. ARRYTH. NOS	arrhythmia
53893	[X]OTHER SPECIFIED CARDIAC ARRHYTHMIAS	arrhythmia
60047	ESSENTIAL PAROXYSMAL TACHYCARDIA	arrhythmia
70366	BOUVERET-HOFFMANN SYNDROME	arrhythmia
83530	PERCUTANEOUS TRANSLUMINAL INTERNAL CARDIOVERSION NEC	arrhythmia
84152	PERC TRANSLUMINAL ABLATION OF ATRIAL WALL FOR ATRIAL FLU	arrhythmia
86416	PERC TRANSLUM ABLAT CONDUCT SYS HEART FOR ATRIAL FLUTTEI	arrhythmia
92361	PERC TRANSLUM ABLAT PULMON VEIN TO LFT ATRIUM CONDUCT	arrhythmia
93460	HISTORY OF ATRIAL FLUTTER	arrhythmia
95919	BRUGADA SYNDROME	arrhythmia
96076	PERSISTENT ATRIAL FIBRILLATION	arrhythmia
96277	Permanent atrial fibrillation	arrhythmia
97780	ARRHYTHMOGENIC RIGHT VENTRICULAR CARDIOMYOPATHY	arrhythmia
107472	Paroxysmal atrial flutter	arrhythmia
109821	ATRIAL STANDSTILL	arrhythmia
7309	[V]Personal history of aspirin allergy	atopy
5768	[V]Personal history of non-drug allergy	atopy
47599	[X]Other allergic rhinitis	atopy
72490	[X]Other seasonal allergic rhinitis	atopy
16676	Acute allergic conjunctivitis	atopy
5869	Allergic (intrinsic) eczema	atopy
2290	Allergic asthma	atopy
334	Allergic contact dermatitis	atopy
7530	Allergic reaction to bee sting	atopy
13401	Allergic reaction to insect bite	atopy
175	Allergic rhinitis	atopy
2372	Allergic rhinitis due to other allergens	atopy
1838	Allergic rhinitis due to pollens	atopy
775	Allergic rhinitis due to unspecified allergen	atopy
964	Allergic rhinitis NOS	atopy
6400	Allergic urticaria	atopy
19862	Allergy skin test positive	atopy
29458	Allergy test positive	atopy
12382	Allergy to animal	atopy
620	Allergy, unspecified	atopy

108904	Atopy	atopy
4861	Cat allergy	atopy
16441	Chronic atrophic rhinitis	atopy
805	Chronic catarrhal rhinitis	atopy
15553	Chronic hypertrophic rhinitis	atopy
774	Chronic rhinitis	atopy
14645	Chronic rhinitis NOS	atopy
28589	Chronic simple rhinitis	atopy
90519	Clinical immunology/allergy	atopy
13377	Dander (animal) allergy	atopy
95938	Dog allergy	atopy
30375	Feather allergy	atopy
489	Food allergy	atopy
11352	H/O: aspirin allergy	atopy
13409	H/O: cat allergy	atopy
13012	H/O: drug allergy NOS	atopy
7418	H/O: food allergy	atopy
9302	H/O: hay fever	atopy
12239	H/O: multiple allergies	atopy
13407	H/O: plant allergy	atopy
16134	Hay fever - other allergen	atopy
121	Hay fever - pollens	atopy
3798	Hay fever - unspecified allergen	atopy
15248	Hay fever with asthma	atopy
5627	Hay fever with asthma	atopy
3162	House dust allergy	atopy
1930	House dust mite allergy	atopy
10182	Nut allergy	atopy
14688	Other chronic allergic conjunctivitis	atopy
4882	Peanut allergy	atopy
1468	Perennial rhinitis	atopy
104108	Referral to clinical allergy service	atopy
3821	Rhinitis - acute	atopy
11306	Seen by clinical allergist	atopy
1108	Vasomotor rhinitis	atopy
1243	O/E - allergic rash	atopy
1275	Allergic reaction	atopy
1973	Allergic drug reaction NOS	atopy
788	Allergic conjunctivitis	atopy
16832	Contact or allergic eyelid dermatitis	atopy
29331	Allergic dis. initial assessm.	atopy
16685	Allergic dermatitis - eyelid	atopy
18572	Allergic rhinosinusitis	atopy
6274	Allergic pharyngitis	atopy
18207	Allergic bronchitis NEC	atopy
43733	Allergic dis. monitoring NOS	atopy
27760	Allergic dis. follow-up assess	atopy
21232	Allergic asthma NEC	atopy
37597	Chronic allergic otitis media	atopy
29845	Allergic otitis media NOS	atopy

53414	Allergic eosinophilia	atopy
47919	Allergic dis.- symptom change	atopy
70788	Acute allergic serous otitis media	atopy
619	skin:type 1 immediate reaction	atopy
1741	atopic dermatitis/eczema	atopy
2011	acute atopic conjunctivitis	atopy
6180	atopic dermatitis nos	atopy
7146	extrinsic (atopic) asthma	atopy
13223	atopic dermatitis and related conditions	atopy
13408	allergic reaction to wasp sting	atopy
30157	under care of clinical allergist	atopy
30664	allergic contact dermatitis due to food in contact with skin	atopy
230	eczema nos	atopy
653	seborrhoeic eczema	atopy
1095	discoid eczema	atopy
1240	flexural eczema	atopy
1424	infected eczema	atopy
1427	contact dermatitis	atopy
2859	h/o: eczema	atopy
3699	hand eczema	atopy
4684	discoid eczema	atopy
5000	pustular eczema	atopy
5391	contact eczema	atopy
6218	eczema of external ear	atopy
6399	contact dermatitis and other eczemas	atopy
15372	pustular eczema	atopy
15879	contact eczema - eyelids	atopy
22764	[x]exacerbation of eczema	atopy
39721	[x]dermatitis and eczema	atopy
296	Cataract	cataract
5361	Other extraction of cataract	cataract
703	Bilateral cataracts	cataract
4260	Intracapsular extraction of cataract	cataract
1622	O/E - cataract present	cataract
6330	Extracapsular extraction of cataract	cataract
6317	Cataract NOS	cataract
103508	Cataract extraction and insertion of intraocular lens	cataract
5518	H/O: cataract	cataract
6547	O/E - Right cataract present	cataract
9931	O/E - Left cataract present	cataract
10010	Senile cataract	cataract
11767	Referral for cataract extraction	cataract
28513	H/O: R cataract extraction	cataract
28515	H/O: L cataract extraction	cataract
46321	Reason for restarting smoking	current smoker
12952	smoking started	current smoker
248526	Pipe smoker	current smoker
101519	[X]Mental and behav dis due to use tobacco: withdrawal state	current smoker
93	cigarette smoker	current smoker
74907	Smoking cessation therapy	current smoker

68658	Tobacco dependence NOS	current smoker
46300	Cigarette pack-years	current smoker
98154	referral to nhs stop smoking service	current smoker
12951	smoking restarted	current smoker
12944	light smoker - 1-9 cigs/day	current smoker
70746	Tobacco dependence, continuous	current smoker
95610	Tobacco dependence, unspecified	current smoker
30762	not interested in stopping smoking	current smoker
9045	advice on smoking	current smoker
294327	Trying to give up smoking	current smoker
257726	Smoker	current smoker
98347	Current smoker annual review - enhanced services admin	current smoker
203207	Smoking restarted	current smoker
32687	Tobacco dependence	current smoker
12947	pipe smoker	current smoker
12943	cigar smoker	current smoker
53101	stop smoking monitor verb.inv.	current smoker
90522	Smoking cessation therapy NOS	current smoker
54	Tobacco consumption	current smoker
40418	refuses stop smoking monitor	current smoker
12941	occasional smoker	current smoker
1823	smoker	current smoker
1822	very heavy smoker - 40+cigs/d	current smoker
41979	smoking restarted	current smoker
1878	moderate smoker - 10-19 cigs/d	current smoker
18926	lifestyle advice regarding smoking	current smoker
12966	smoking reduced	current smoker
31114	ready to stop smoking	current smoker
104310	current smoker annual review	current smoker
12945	rolls own cigarettes	current smoker
203208	Current smoker	current smoker
276050	Cigar smoker	current smoker
10742	referral to stop-smoking clinic	current smoker
309558	Smoking reduced	current smoker
12964	Keeps trying to stop smoking	current smoker
10558	current smoker	current smoker
257725	Keeps trying to stop smoking	current smoker
11713	Pack years	current smoker
42288	Pack years	current smoker
2111	health ed. - smoking	current smoker
35055	[V]Tobacco abuse counselling	current smoker
10184	pregnancy smoking advice	current smoker
12958	trivial smoker - < 1 cig/day	current smoker
12954	[V]Tobacco use	current smoker
3568	heavy smoker - 20-39 cigs/day	current smoker
12965	cigarette consumption	current smoker
41042	Smoking cessation advice provided by community pharmacist	current smoker
266944	Rolls own cigarettes	current smoker
12960	Tobacco consumption NOS	current smoker
12240	trying to give up smoking	current smoker

30423	thinking about stopping smoking	current smoker
276051	Smoking started	current smoker
285187	Cigarette smoker	current smoker
12967	Pipe tobacco consumption	current smoker
12963	cigar consumption	current smoker
26096	smokes drugs	current smoker
12942	smoker - amount smoked	current smoker
324	Depressive disorder NEC	depression
543	[X]Depression NOS	depression
595	Endogenous depression	depression
655	Anxiety with depression	depression
1055	Agitated depression	depression
1131	Neurotic depression reactive type	depression
1533	Brief depressive reaction	depression
2560	Depressive psychoses	depression
2639	Postnatal depression	depression
2923	Puerperal depression	depression
2970	[X]Depressive episode, unspecified	depression
2972	Postviral depression	depression
3291	[X]Depressive disorder NOS	depression
3292	[X]Recurrent depressive disorder	depression
4323	Chronic depression	depression
4639	[X]Depressive episode	depression
4979	[X]Postpartum depression NOS	depression
5879	Agitated depression	depression
5987	[X] Reactive depression NOS	depression
6482	Recurrent depression	depression
6546	Endogenous depression first episode	depression
6854	[X]Other depressive episodes	depression
6932	Endogenous depression - recurrent	depression
6950	Endogenous depression first episode	depression
7011	Single major depressive episode NOS	depression
7604	[X]Single episode of reactive depression	depression
7737	[X]Neurotic depression	depression
7749	[X]Mild anxiety depression	depression
7953	[X]Dysthymia	depression
8478	Reactive depressive psychosis	depression
8584	[X]Depressive neurosis	depression
8826	[X]SAD - Seasonal affective disorder	depression
8851	[X]Recurrent episodes of depressive reaction	depression
8902	[X]Recurrent episodes of reactive depression	depression
9055	[X]Single episode of depressive reaction	depression
9183	Masked depression	depression
9211	[X]Moderate depressive episode	depression
9667	[X]Severe depressive episode without psychotic symptoms	depression
10290	[X]Depressive personality disorder	depression
10455	Depressive personality disorder	depression
10610	Single major depressive episode	depression
10667	[X]Mild depression	depression
10720	[X]Atypical depression	depression

10825	Seasonal affective disorder	depression
11055	[X]Schizoaffective disorder, depressive type	depression
11252	[X]Major depression, recurrent without psychotic symptoms	depression
11329	[X]Endogenous depression without psychotic symptoms	depression
11717	[X]Mild depressive episode	depression
11913	[X]Mixed anxiety and depressive disorder	depression
12099	[X]Severe depressive episode with psychotic symptoms	depression
12122	Depression medication review	depression
12399	Depression annual review	depression
13307	[X]Postnatal depression NOS	depression
14709	Recurrent major depressive episodes, moderate	depression
15099	Recurrent major depressive episode	depression
15155	Single major depressive episode, moderate	depression
15219	Single major depressive episode, severe, without psychosis	depression
15220	[X]Persistant anxiety depression	depression
16506	Single major depressive episode, mild	depression
16632	Prolonged depressive reaction	depression
16861	[X]Recurrent severe episodes of psychotic depression	depression
17770	Psychotic reactive depression	depression
18510	[X]Single episode of psychogenic depression	depression
19054	[X]Recurrent brief depressive episodes	depression
19696	[X]Recurrent episodes of psychogenic depression	depression
20785	[X]Post-schizophrenic depression	depression
21887	Senile dementia with depression	depression
22806	[X]Single episode major depression w'out psychotic symptoms	depression
23731	[X]Endogenous depression with psychotic symptoms	depression
24112	[X]Single episode of psychotic depression	depression
24117	[X]Single episode of major depression and psychotic symptoms	depression
24171	Recurrent major depressive episodes, severe, with psychosis	depression
25563	Recurrent major depressive episode NOS	depression
25697	Recurrent major depressive episodes, severe, no psychosis	depression
27491	Atypical depressive disorder	depression
27677	Presenile dementia with depression	depression
27759	[X] Senile dementia, depressed or paranoid type	depression
28248	[X]Prolonged single episode of reactive depression	depression
28677	[X]Manic-depress psychosis,depressed type+psychotic symptoms	depression
28756	[X]Seasonal depressive disorder	depression
28863	[X]Single episode of reactive depressive psychosis	depression
29342	Recurrent major depressive episodes, mild	depression
29451	[X]Manic-depress psychosis,depressd,no psychotic symptoms	depression
29520	[X]Recurrent depressive disorder, current episode moderate	depression
29527	[D]Postoperative depression	depression
29784	[X]Recurrent depressive disorder, current episode mild	depression
30405	Depression interim review	depression
31757	[X]Recurr severe episodes/psychogenic depressive psychosis	depression
32159	Single major depressive episode, severe, with psychosis	depression
32941	[X]Recurr severe episodes/major depression+psychotic symptom	depression
33469	[X]Recurr depress disorder cur epi severe without psyc sympt	depression
34390	Single major depressive episode, unspecified	depression
35274	[X]Schizoaffective psychosis, depressive type	depression

35671	Recurrent major depressive episodes, unspecified	depression
36246	Brief depressive reaction NOS	depression
36616	[X]Monopolar depression NOS	depression
37764	[X]Recurrent severe episodes/reactive depressive psychosis	depression
41022	[X]Schizophreniform psychosis, depressive type	depression
41089	Senile dementia with depressive or paranoid features NOS	depression
41989	[X]Single episode agitated depressn w'out psychotic symptoms	depression
43292	Arteriosclerotic dementia with depression	depression
44300	[X]Recurrent depressive disorder, unspecified	depression
44674	Senile dementia with depressive or paranoid features	depression
44848	Depression management programme	depression
47009	[X]Recurrent depress disorder cur epi severe with psyc symp	depression
47731	[X]Other recurrent depressive disorders	depression
52678	[X]Single episode of psychogenic depressive psychosis	depression
56609	[X]Single episode of masked depression NOS	depression
59386	[X]Single episode vital depression w'out psychotic symptoms	depression
73991	[X]Vital depression, recurrent without psychotic symptoms	depression
98252	[X]Major depression, moderately severe	depression
98346	[X]Major depression, mild	depression
98414	[X]Major depression, severe without psychotic symptoms	depression
98417	[X]Major depression, severe with psychotic symptoms	depression
6125	Diabetic annual review	diabetes
2379	Seen in diabetic clinic	diabetes
758	Type 2 diabetes mellitus	diabetes
711	Diabetes mellitus	diabetes
11471	Diabetes medication review	diabetes
1684	Diabetic on oral treatment	diabetes
8836	Diabetes management plan given	diabetes
2378	Diabetic - poor control	diabetes
7563	Diabetic on diet only	diabetes
28873	Diabetic 6 month review	diabetes
12213	Patient on maximal tolerated therapy for diabetes	diabetes
1323	Diabetic retinopathy	diabetes
506	Non-insulin dependent diabetes mellitus	diabetes
1549	Type 1 diabetes mellitus	diabetes
4513	Non-insulin dependent diabetes mellitus	diabetes
83532	Diabetes type 2 review	diabetes
8842	Diabetic on insulin	diabetes
12675	Diabetes: shared care programme	diabetes
13071	Diabetic - good control	diabetes
17859	Type 2 diabetes mellitus	diabetes
1038	Insulin dependent diabetes mellitus	diabetes
3837	Diabetic maculopathy	diabetes
1647	Insulin dependent diabetes mellitus	diabetes
14889	Maturity onset diabetes	diabetes
28769	Diabetic on insulin and oral treatment	diabetes
14803	Diabetes mellitus, adult onset, no mention of complication	diabetes
10755	Non proliferative diabetic retinopathy	diabetes
1682	Diabetes mellitus with ketoacidosis	diabetes
1407	Insulin treated Type 2 diabetes mellitus	diabetes

16230	Diabetes mellitus with neurological manifestation	diabetes
3286	Proliferative diabetic retinopathy	diabetes
7795	Diabetes mellitus with neuropathy	diabetes
18390	Type 2 diabetes mellitus with persistent microalbuminuria	diabetes
2342	Diabetic neuropathy	diabetes
18505	IDDM-Insulin dependent diabetes mellitus	diabetes
17858	Type 1 diabetes mellitus	diabetes
5884	NIDDM - Non-insulin dependent diabetes mellitus	diabetes
10692	Type 1 diabetes mellitus with ketoacidosis	diabetes
22884	Type II diabetes mellitus	diabetes
18219	Type II diabetes mellitus	diabetes
2986	Preproliferative diabetic retinopathy	diabetes
26054	Type 2 diabetes mellitus with persistent proteinuria	diabetes
18278	Insulin treated Type 2 diabetes mellitus	diabetes
35399	Diabetes mellitus with peripheral circulatory disorder	diabetes
2475	Diabetic nephropathy	diabetes
38986	Diabetes mellitus with no mention of complication	diabetes
11626	Diabetic retinopathy NOS	diabetes
18496	Type 2 diabetes mellitus with retinopathy	diabetes
8403	Non-insulin dependant diabetes mellitus - poor control	diabetes
22023	Diabetic - poor control NOS	diabetes
16502	Diabetes mellitus with renal manifestation	diabetes
25627	Type 2 diabetes mellitus - poor control	diabetes
51261	Insulin dependent diabetes mellitus	diabetes
9013	Unstable diabetes	diabetes
47954	Type 2 diabetes mellitus without complication	diabetes
32627	Type 2 diabetes mellitus with ketoacidosis	diabetes
24423	Type I diabetes mellitus	diabetes
10099	Advanced diabetic maculopathy	diabetes
12455	Type I diabetes mellitus	diabetes
50972	Diabetes mellitus NOS with no mention of complication	diabetes
34450	Hyperosmolar non-ketotic state in type 2 diabetes mellitus	diabetes
31310	Insulin dependant diabetes maturity onset	diabetes
18387	Type 1 diabetes mellitus with retinopathy	diabetes
30323	Type 1 diabetes mellitus with persistent proteinuria	diabetes
10418	Type 1 diabetes mellitus with nephropathy	diabetes
30294	Type 1 diabetes mellitus with persistent microalbuminuria	diabetes
6791	Insulin dependant diabetes mellitus - poor control	diabetes
42505	Diabetes mellitus NOS with ketoacidosis	diabetes
15690	Diabetes mellitus with ketoacidotic coma	diabetes
35288	Type 1 diabetes mellitus - poor control	diabetes
36695	Diabetes mellitus autosomal dominant type 2	diabetes
55239	Type 1 diabetes mellitus with gastroparesis	diabetes
37315	Diabetic mononeuropathy	diabetes
35107	Diabetes mellitis with nephropathy NOS	diabetes
32403	Diabetes mellitus with gangrene	diabetes
29979	Non-insulin-dependent diabetes mellitus without complication	diabetes
18425	Type 2 diabetes mellitus with polyneuropathy	diabetes
53392	Type II diabetes mellitus without complication	diabetes
22573	Diabetes mellitus NOS with neurological manifestation	diabetes

6509	Insulin dependent diabetes mellitus with retinopathy	diabetes
40837	Type 1 diabetes mellitus with ketoacidotic coma	diabetes
18777	Type 2 diabetes mellitus with renal complications	diabetes
34912	Non-insulin dependent diabetes mellitus with ulcer	diabetes
46624	Maturity onset diabetes in youth	diabetes
35385	Type 2 diabetes mellitus with neuropathic arthropathy	diabetes
21482	Diabetes mellitus with hyperosmolar coma	diabetes
44443	Insulin dependent diabetes mellitus with ulcer	diabetes
52212	[X]Diabetes mellitus	diabetes
39317	Diabetes mellitus, adult onset, + neurological manifestation	diabetes
25591	Type 2 diabetes mellitus with exudative maculopathy	diabetes
63690	Type 2 diabetes mellitus with gastroparesis	diabetes
34268	Type 2 diabetes mellitus with neurological complications	diabetes
34283	Diabetes mellitus NOS with ophthalmic manifestation	diabetes
22487	Secondary diabetes mellitus	diabetes
55431	Pre-existing diabetes mellitus, unspecified	diabetes
44982	Type 2 diabetes mellitus with diabetic cataract	diabetes
49074	Type 2 diabetes mellitus with ulcer	diabetes
17262	Non-insulin-dependent diabetes mellitus with retinopathy	diabetes
64357	Diabetes mellitus NOS with unspecified complication	diabetes
33969	Malnutrition-related diabetes mellitus with ketoacidosis	diabetes
95094	Did not complete diabetes structured education programme	diabetes
64668	Insulin treated Type II diabetes mellitus	diabetes
35105	Diabetes mellitus, adult onset, with renal manifestation	diabetes
47315	Type II diabetes mellitus - poor control	diabetes
26855	Unstable insulin dependant diabetes mellitus	diabetes
41389	Diabetes mellitus, adult onset, + ophthalmic manifestation	diabetes
45491	Diabetes mellitus with unspecified complication	diabetes
37806	Type 2 diabetes mellitus with peripheral angiopathy	diabetes
39070	Type 1 diabetes mellitus with hypoglycaemic coma	diabetes
65025	Diabetes mellitus NOS with peripheral circulatory disorder	diabetes
67853	Diabetes mellitus, juvenile, + neurological manifestation	diabetes
33343	Diabetes mellitus with other specified manifestation	diabetes
62674	Type 2 diabetes mellitus with mononeuropathy	diabetes
16491	Diabetes mellitus with polyneuropathy	diabetes
22871	Type 1 diabetes mellitus with exudative maculopathy	diabetes
59365	Non-insulin dependent diabetes mellitus with nephropathy	diabetes
12640	Type 2 diabetes mellitus with nephropathy	diabetes
18264	Insulin treated Type II diabetes mellitus	diabetes
54008	Type 1 diabetes mellitus with neuropathic arthropathy	diabetes
60796	Type II diabetes mellitus with persistent proteinuria	diabetes
61523	Other specified diabetes mellitus with neurological comps	diabetes
93727	Type II diabetes mellitus with diabetic cataract	diabetes
12736	Type 2 diabetes mellitus with gangrene	diabetes
108005	Type 2 diabetes mellitus with multiple complications	diabetes
63357	Diabetes mellitus, adult, + peripheral circulatory disorder	diabetes
40682	Type 1 diabetes mellitus maturity onset	diabetes
63762	Diabetes mellitus, adult onset, + unspecified complication	diabetes
69676	Type 1 diabetes mellitus without complication	diabetes
44440	Insulin dependent diabetes mellitus with hypoglycaemic coma	diabetes

47582	Type 1 diabetes mellitus with renal complications	diabetes
59253	Type 2 diabetes mellitus with arthropathy	diabetes
24458	Type II diabetes mellitus - poor control	diabetes
43921	Unstable type 1 diabetes mellitus	diabetes
45913	Type 2 diabetes mellitus - poor control	diabetes
62209	Type I diabetes mellitus with ketoacidosis	diabetes
18683	Type 1 diabetes mellitus with ulcer	diabetes
33807	Diabetes mellitus, adult with gangrene	diabetes
50609	Pre-existing diabetes mellitus, non-insulin-dependent	diabetes
54856	Diabetes mellitus, adult onset, with ketoacidosis	diabetes
56803	NIDDM with peripheral circulatory disorder	diabetes
36633	Hyperosmolar non-ketotic state in type 2 diabetes mellitus	diabetes
102201	Type II diabetes mellitus with nephropathy	diabetes
49655	Type II diabetes mellitus with retinopathy	diabetes
70316	Type 2 diabetes mellitus with ophthalmic complications	diabetes
65267	Type 2 diabetes mellitus with multiple complications	diabetes
47650	Type 1 diabetes mellitus with multiple complications	diabetes
65463	High risk non proliferative diabetic retinopathy	diabetes
43453	Diabetes mellitus autosomal dominant	diabetes
46301	Type 1 diabetes mellitus with polyneuropathy	diabetes
38617	Other specified diabetes mellitus with ketoacidosis	diabetes
59903	Diabetic amyotrophy	diabetes
51756	Type 2 diabetes mellitus with ketoacidotic coma	diabetes
59991	Maturity onset diabetes in youth type 2	diabetes
46963	Insulin-dependent diabetes mellitus with renal complications	diabetes
39809	Insulin dependent diab mell with neuropathic arthropathy	diabetes
104639	Type II diabetes mellitus with peripheral angiopathy	diabetes
37648	Insulin treated non-insulin dependent diabetes mellitus	diabetes
42762	Type 2 diabetes mellitus with retinopathy	diabetes
42831	Type 1 diabetes mellitus with neurological complications	diabetes
43139	Diabetes mellitus, adult onset, with hyperosmolar coma	diabetes
98723	Type II diabetes mellitus with hypoglycaemic coma	diabetes
18209	Type 2 diabetes mellitus with renal complications	diabetes
47649	Type 1 diabetes mellitus with ophthalmic complications	diabetes
56448	Insulin-dependent diabetes without complication	diabetes
58604	Type II diabetes mellitus with retinopathy	diabetes
49554	Type 1 diabetes mellitus with diabetic cataract	diabetes
50527	Type II diabetes mellitus with polyneuropathy	diabetes
55075	Type II diabetes mellitus with ulcer	diabetes
46850	Type I diabetes mellitus - poor control	diabetes
49276	Insulin-dependent diabetes mellitus with ophthalmic comps	diabetes
57621	Insulin dependent diabetes mellitus with nephropathy	diabetes
69278	Non-insulin depend diabetes mellitus with diabetic cataract	diabetes
103902	Type II diabetes mellitus with arthropathy	diabetes
52303	Non-insulin-dependent diabetes mellitus with renal comps	diabetes
95343	Type I diabetes mellitus with retinopathy	diabetes
45276	Insulin dependent diabetes mellitus with multiple complications	diabetes
45919	Type 2 diabetes mellitus with neurological complications	diabetes
55842	Non-insulin-dependent diabetes mellitus with neuro comps	diabetes
72702	Insulin dependent diabetes mellitus - poor control	diabetes

45467	Non-insulin dependent diabetes mellitus with polyneuropathy	diabetes
68390	Type 1 diabetes mellitus with ulcer	diabetes
69124	IDDM with peripheral circulatory disorder	diabetes
70821	Diabetes mellitus NOS with other specified manifestation	diabetes
13279	Other specified diabetes mellitus with renal complications	diabetes
38161	Type I diabetes mellitus with retinopathy	diabetes
40401	Non-insulin dependent diabetes mellitus with gangrene	diabetes
42729	Type I diabetes mellitus with hypoglycaemic coma	diabetes
48192	Type II diabetes mellitus with diabetic cataract	diabetes
65704	Type 2 diabetes mellitus with ulcer	diabetes
96235	Type I diabetes mellitus maturity onset	diabetes
97849	Insulin dependent diabetes mellitus maturity onset	diabetes
65062	Diabetes mellitus NOS with ketoacidotic coma	diabetes
64283	Other specified diabetes mellitus with unspecified comps	diabetes
69993	Type 1 diabetes mellitus with gangrene	diabetes
18642	Type 1 diabetes mellitus with arthropathy	diabetes
40962	Non-insulin dependent d m with neuropathic arthropathy	diabetes
41716	Insulin dependent diabetes mellitus with polyneuropathy	diabetes
52104	Insulin dependent diabetes mellitus with multiple complicatn	diabetes
68105	Type 1 diabetes mellitus with mononeuropathy	diabetes
95351	Type II diabetes mellitus with mononeuropathy	diabetes
24836	Type 2 diabetes mellitus with nephropathy	diabetes
43785	Non-insulin dependent diabetes mellitus with hypoglyca coma	diabetes
46150	Type 2 diabetes mellitus with gangrene	diabetes
51957	Type I diabetes mellitus with ulcer	diabetes
60499	Insulin dependent diabetes mellitus with gangrene	diabetes
40023	Diabetes mellitus, juvenile type, with hyperosmolar coma	diabetes
41049	Type 1 diabetes mellitus with retinopathy	diabetes
43227	Type II diabetes mellitus with multiple complications	diabetes
44779	Type 2 diabetes mellitus with diabetic cataract	diabetes
45914	Type 1 diabetes mellitus - poor control	diabetes
49949	Unstable type I diabetes mellitus	diabetes
50225	Type II diabetes mellitus with renal complications	diabetes
52283	Insulin-dependent diabetes mellitus with neurological comps	diabetes
54212	Non-insulin-dependent d m with peripheral angiopath	diabetes
62146	Non-insulin-dependent diabetes mellitus with multiple comps	diabetes
66965	Type 2 diabetes mellitus with neuropathic arthropathy	diabetes
10098	Other specified diabetes mellitus with other spec comps	diabetes
44260	Insulin dependent diabetes mellitus with diabetic cataract	diabetes
47816	Type II diabetes mellitus with neuropathic arthropathy	diabetes
61071	Type 2 diabetes mellitus with hypoglycaemic coma	diabetes
63371	Diabetes mellitus, adult, + other specified manifestation	diabetes
93875	Insulin dependent diabetes mellitus with retinopathy	diabetes
94383	Secondary diabetes mellitus without complication	diabetes
102112	Type I diabetes mellitus with gangrene	diabetes
105337	Type I diabetes mellitus - poor control	diabetes
42567	Diabetes mellitus, juvenile type, with ketoacidotic coma	diabetes
47377	Other specified diabetes mellitus with ophthalmic complicatn	diabetes
62107	Type II diabetes mellitus with gangrene	diabetes
66872	Type I diabetes mellitus with nephropathy	diabetes

68792	Diabetes mellitus, juvenile type, + unspecified complication	diabetes
72345	Diabetes mellitus NOS with hyperosmolar coma	diabetes
95539	Maternally inherited diabetes mellitus	diabetes
98392	Maturity onset diabetes in youth type 1	diabetes
54600	Unstable insulin dependent diabetes mellitus	diabetes
61344	Type I diabetes mellitus with renal complications	diabetes
61829	Type 1 diabetes mellitus with neurological complications	diabetes
69748	Diabetes mellitus, juvenile type, + ophthalmic manifestation	diabetes
93468	Type 1 diabetes mellitus with peripheral angiopathy	diabetes
93878	Type I diabetes mellitus with ulcer	diabetes
17545	Type I diabetes mellitus with diabetic cataract	diabetes
21983	Type 1 diabetes mellitus with renal complications	diabetes
41686	[X]Other specified diabetes mellitus	diabetes
57278	Type II diabetes mellitus with renal complications	diabetes
64571	Type II diabetes mellitus with nephropathy	diabetes
67905	Type II diabetes mellitus with neurological complications	diabetes
91646	Type II diabetes mellitus with ulcer	diabetes
91942	Type I diabetes mellitus with multiple complications	diabetes
93922	Diabetes mellitus, juvenile type, with renal manifestation	diabetes
100964	Type II diabetes mellitus with ophthalmic complications	diabetes
106528	Type II diabetes mellitus with ketoacidosis	diabetes
24693	Non-insulin dependent diabetes mellitus with arthropathy	diabetes
47409	Type II diabetes mellitus with polyneuropathy	diabetes
54899	Type II diabetes mellitus with peripheral angiopathy	diabetes
60107	Unstable type I diabetes mellitus	diabetes
60699	Type 2 diabetes mellitus with peripheral angiopathy	diabetes
62613	Type I diabetes mellitus without complication	diabetes
65616	Insulin dependent diabetes mellitus with arthropathy	diabetes
68843	Diabetes mellitus, adult onset, with ketoacidotic coma	diabetes
85991	Type II diabetes mellitus with persistent microalbuminuria	diabetes
102946	Insulin-dependent diabetes mellitus with renal complications	diabetes
109197	Type II diabetes mellitus with neuropathic arthropathy	diabetes
18230	Type 1 diabetes mellitus with neuropathic arthropathy	diabetes
59288	Other specified diabetes mellitus with coma	diabetes
60208	Type I diabetes mellitus with neuropathic arthropathy	diabetes
64446	Insulin dependent diab mell with peripheral angiopathy	diabetes
64449	Unspecified diabetes mellitus with multiple complications	diabetes
66675	Malnutrition-related diabetes mellitus with coma	diabetes
70766	Type 1 diabetes mellitus with hypoglycaemic coma	diabetes
72320	Non-insulin dependent diabetes mellitus with mononeuropathy	diabetes
97474	Unstable type 1 diabetes mellitus	diabetes
98704	Insulin dependent diabetes mellitus with ulcer	diabetes
104323	Type II diabetes mellitus with gangrene	diabetes
105784	Type 2 diabetes mellitus without complication	diabetes
109865	Type 2 diabetes mellitus with polyneuropathy	diabetes
24694	Insulin dependent diabetes mellitus with mononeuropathy	diabetes
56268	Type II diabetes mellitus with hypoglycaemic coma	diabetes
59725	Type II diabetes mellitus with ophthalmic complications	diabetes
70448	Diabetes mellitus, juvenile +peripheral circulatory disorder	diabetes
96506	Secondary pancreatic diabetes mellitus without complication	diabetes

97446	Type 1 diabetes mellitus maturity onset	diabetes
97894	Type I diabetes mellitus with exudative maculopathy	diabetes
98071	Insulin-dependent diabetes mellitus with ophthalmic complication:	diabetes
98616	Type II diabetes mellitus with neurological complications	diabetes
99716	Insulin dependent diabetes mellitus with hypoglycaemic coma	diabetes
99719	Insulin-dependent diabetes mellitus without complication	diabetes
100292	[X]Unspecified diabetes mellitus with renal complications	diabetes
101311	Insulin dependent diabetes mellitus with polyneuropathy	diabetes
106927	Diabetes insipidus, diabetes mellitus, optic atrophy and deafness	diabetes
107701	Hyperosmolar non-ketotic state in type II diabetes mellitus	diabetes
18143	Type II diabetes mellitus with arthropathy	diabetes
49146	Type I diabetes mellitus with neurological complications	diabetes
49869	Type 2 diabetes mellitus with arthropathy	diabetes
50813	Type II diabetes mellitus with mononeuropathy	diabetes
62352	Type I diabetes mellitus with arthropathy	diabetes
63017	Type I diabetes mellitus maturity onset	diabetes
66145	Type I diabetes mellitus with ketoacidotic coma	diabetes
91943	Type I diabetes mellitus with polyneuropathy	diabetes
95992	Type I diabetes mellitus without complication	diabetes
99231	Type I diabetes mellitus with mononeuropathy	diabetes
99311	Type I diabetes mellitus with ophthalmic complications	diabetes
100347	Malnutritn-relat diabetes melitus wth periph circul complctn	diabetes
100770	Insulin dependent diabetes mellitus with diabetic cataract	diabetes
101735	Insulin-depended diabetes mellitus with neurological comps	diabetes
102163	Insulin dependent diabetes mellitus with nephropathy	diabetes
102620	Type I diabetes mellitus with persistent microalbuminuria	diabetes
102740	Type 1 diabetes mellitus with ophthalmic complications	diabetes
106061	Type II diabetes mellitus with ketoacidotic coma	diabetes
108007	Type I diabetes mellitus with multiple complications	diabetes
108724	Type I diabetes mellitus with gastroparesis	diabetes
109051	Insulin dependent diabetes mellitus with gangrene	diabetes
109133	Pre-existing malnutrition-related diabetes mellitus	diabetes
109837	Type I diabetes mellitus with renal complications	diabetes
110400	Type 1 diabetes mellitus with diabetic cataract	diabetes
111798	Type II diabetes mellitus with exudative maculopathy	diabetes
109103	Type II diabetes mellitus without complication	diabetes
97210	Ex-cigarette smoker	exsmoker
12946	ex-smoker - amount unknown	exsmoker
230314	Ex-smoker - amount unknown	exsmoker
221248	Ex-very heavy smoker (40+/day)	exsmoker
276052	Date ceased smoking	exsmoker
99838	recently stopped smoking	exsmoker
72700	[V]Personal history of tobacco abuse	exsmoker
7130	stop smoking monitoring admin.	exsmoker
102361	Referral for smoking cessation service offered	exsmoker
12878	date ceased smoking	exsmoker
266945	Ex pipe smoker	exsmoker
12955	ex-moderate smoker (10-19/day)	exsmoker
43433	Toxic effect of tobacco and nicotine	exsmoker
776	stopped smoking	exsmoker

12956	Ex-heavy smoker (20-39/day)	exsmoker
97973	Maternal tobacco abuse	exsmoker
98447	ex-smoker annual review - enhanced services administration	exsmoker
72706	Tobacco dependence in remission	exsmoker
19488	ex cigar smoker	exsmoker
26470	ex pipe smoker	exsmoker
100963	ex-smoker annual review	exsmoker
12961	ex-trivial smoker (<1/day)	exsmoker
90	Ex smoker	exsmoker
12959	Ex-very heavy smoker (40+/day)	exsmoker
12957	ex-light smoker (1-9/day)	exsmoker
239315	Ex-heavy smoker (20-39/day)	exsmoker
10898	Smoking free weeks	exsmoker
248528	Stopped smoking	exsmoker
101338	Failed attempt to stop smoking	exsmoker
294328	Ex cigar smoker	exsmoker
103955	Asthma trigger - tobacco smoke	exsmoker
100099	Smoking cessation advice declined	exsmoker
100495	ex roll-up cigarette smoker	exsmoker
62686	Minutes from waking to first tobacco consumption	exsmoker
7438	[X]Osteopenia	osteopenia
28882	Referral to osteoporosis clinic	osteoporosis
26876	Osteoporosis - dietary advice	osteoporosis
10359	Follow-up osteoporosis assessment	osteoporosis
96779	Osteoporosis monitoring deleted	osteoporosis
38395	Postmenopausal osteoporosis with pathological fracture	osteoporosis
16857	Osteoporosis localized to spine	osteoporosis
60618	Osteoporosis risk assessment refused	osteoporosis
33526	Osteoporosis of disuse with pathological fracture	osteoporosis
68019	Postsurgical malabsorption osteoporosis with path fracture	osteoporosis
37646	Osteoporosis treatment started	osteoporosis
34798	Osteoporosis NOS	osteoporosis
60433	Osteoporosis in multiple myelomatosis	osteoporosis
14967	Osteoporosis, unspecified	osteoporosis
93655	Postsurgical malabsorption osteoporosis	osteoporosis
18265	Seen in osteoporosis clinic	osteoporosis
18825	[X]Unspecified osteoporosis with pathological fracture	osteoporosis
45736	Collapse of cervical vertebra due to osteoporosis	osteoporosis
12673	Osteoporosis + pathological fracture thoracic vertebrae	osteoporosis
48772	Osteoporosis + pathological fracture cervical vertebrae	osteoporosis
25650	Osteoporosis due to corticosteroids	osteoporosis
57301	[X]Other osteoporosis with pathological fracture	osteoporosis
105290	Osteoporosis monitoring verbal invitation	osteoporosis
41376	Osteoporosis - exercise advice	osteoporosis
98433	Osteoporosis - enhanced services administration	osteoporosis
101386	Osteoporosis resolved	osteoporosis
16307	Senile osteoporosis	osteoporosis
68122	Osteoporosis monitoring first letter	osteoporosis
277	Osteoporosis	osteoporosis
39596	Refer to osteoporosis specialist	osteoporosis

93455	Osteoporosis monitoring second letter	osteoporosis
24959	Osteoporosis risk assessment done	osteoporosis
65163	Attends osteoporosis monitoring	osteoporosis
102169	Exception reporting: osteoporosis quality indicators	osteoporosis
17377	Osteoporosis + pathological fracture lumbar vertebrae	osteoporosis
41755	[X]Other osteoporosis	osteoporosis
101068	Osteoporosis medication prophylaxis	osteoporosis
93705	Minimal trauma fracture due to unspecified osteoporosis	osteoporosis
61121	Osteoporosis monitoring telephone invitation	osteoporosis
36644	Osteoporosis treatment stopped	osteoporosis
48962	Osteoporosis - no treatment	osteoporosis
38903	Osteoporosis - dietary assessment	osteoporosis
26292	Osteoporosis - falls prevention	osteoporosis
89922	Osteoporosis treatment not indicated	osteoporosis
98760	Bone sparing drug treatment offered for osteoporosis - ESA	osteoporosis
99817	History of osteoporosis	osteoporosis
27597	Idiopathic osteoporosis with pathological fracture	osteoporosis
104186	Excepted osteoporosis quality indicators: informed dissent	osteoporosis
45274	Refuses osteoporosis monitoring	osteoporosis
39334	Postoophorectomy osteoporosis with pathological fracture	osteoporosis
40428	Idiopathic osteoporosis	osteoporosis
92887	Osteoporosis monitoring default	osteoporosis
9700	Postmenopausal osteoporosis	osteoporosis
11218	FH: Osteoporosis	osteoporosis
11503	Fragility fracture due to unspecified osteoporosis	osteoporosis
101443	Excepted osteoporosis quality indicators: patient unsuitable	osteoporosis
62702	Dissuse osteoporosis	osteoporosis
60949	Osteoporosis risk assessment defaulted	osteoporosis
10362	At risk of osteoporosis	osteoporosis
19048	Collapse of thoracic vertebra due to osteoporosis	osteoporosis
4013	Collapse of vertebra due to osteoporosis NOS	osteoporosis
10293	Initial osteoporosis assessment	osteoporosis
98189	Osteoporosis - no treatment response	osteoporosis
25534	Osteoporosis monitoring check done	osteoporosis
31580	Osteoporosis in endocrine disorders	osteoporosis
13055	Health education - osteoporosis	osteoporosis
24093	Drug-induced osteoporosis	osteoporosis
70349	Postoophorectomy osteoporosis	osteoporosis
34129	Osteoporosis treatment changed	osteoporosis
5841	Collapse of lumbar vertebra due to osteoporosis	osteoporosis
102730	[X]Osteoporosis in other disorders classified elsewhere	osteoporosis
46894	Drug-induced osteoporosis with pathological fracture	osteoporosis
110401	Osteoporosis medication compliance review	osteoporosis
70233	Osteoporosis - treatment response	osteoporosis
36796	Osteoporosis monitoring administration	osteoporosis
54232	Localized osteoporosis - Lequesne	osteoporosis
3346	Vertebral osteoporosis	osteoporosis
11603	Osteoporosis monitoring	osteoporosis
102017	Osteoporosis monitoring third letter	osteoporosis
58896	Salmonella pneumonia	pneumonia

25462	Varicella pneumonitis	pneumonia
47973	Herpes simplex pneumonia	pneumonia
32172	Postmeasles pneumonia	pneumonia
50408	Ornithosis with pneumonia	pneumonia
45072	Cytomegaloviral pneumonitis	pneumonia
40299	Pneumonia - candidal	pneumonia
101507	Histoplasma capsulatum with pneumonia	pneumonia
101292	Histoplasma duboisii with pneumonia	pneumonia
56762	Toxoplasma pneumonitis	pneumonia
10086	Pneumonia and influenza	pneumonia
5202	Viral pneumonia	pneumonia
9389	Chest infection - viral pneumonia	pneumonia
67836	Pneumonia due to adenovirus	pneumonia
31269	Pneumonia due to respiratory syncytial virus	pneumonia
36675	Pneumonia due to parainfluenza virus	pneumonia
33478	Viral pneumonia NEC	pneumonia
14976	Viral pneumonia NOS	pneumonia
1849	Lobar (pneumococcal) pneumonia	pneumonia
29166	Chest infection - pneumococcal pneumonia	pneumonia
28634	Other bacterial pneumonia	pneumonia
22795	Chest infection - other bacterial pneumonia	pneumonia
23546	Pneumonia due to klebsiella pneumoniae	pneumonia
30591	Pneumonia due to pseudomonas	pneumonia
37881	Pneumonia due to haemophilus influenzae	pneumonia
48804	Pneumonia due to haemophilus influenzae	pneumonia
12423	Pneumonia due to streptococcus	pneumonia
63858	Pneumonia due to streptococcus, group B	pneumonia
5612	Pneumonia due to staphylococcus	pneumonia
50867	Pneumonia due to other specified bacteria	pneumonia
65419	Pneumonia due to escherichia coli	pneumonia
60299	E.coli pneumonia	pneumonia
45425	Pneumonia due to proteus	pneumonia
12061	Pneumonia - Legionella	pneumonia
52384	Pneumonia due to other aerobic gram-negative bacteria	pneumonia
43884	Pneumonia due to bacteria NOS	pneumonia
23095	Bacterial pneumonia NOS	pneumonia
25694	Pneumonia due to other specified organisms	pneumonia
30653	Chest infection - pneumonia organism OS	pneumonia
60119	Pneumonia due to Eaton's agent	pneumonia
1576	Pneumonia due to mycoplasma pneumoniae	pneumonia
73735	Pneumonia due to pleuropneumonia like organisms	pneumonia
17025	Chlamydial pneumonia	pneumonia
34251	Pneumonia due to specified organism NOS	pneumonia
40498	Pneumonia with infectious diseases EC	pneumonia
41034	Pneumonia with measles	pneumonia
43286	Pneumonia with cytomegalic inclusion disease	pneumonia
62623	Pneumonia with ornithosis	pneumonia
30437	Pneumonia with whooping cough	pneumonia
35082	Pneumonia with pertussis	pneumonia
34274	Pneumonia with aspergillosis	pneumonia

52071	Pneumonia with candidiasis	pneumonia
53969	Pneumonia with systemic mycosis NOS	pneumonia
69782	Pneumonia with other infectious diseases EC	pneumonia
61623	Pneumonia with actinomycosis	pneumonia
67901	Pneumonia with nocardiasis	pneumonia
27519	Pneumonia with pneumocystis carinii	pneumonia
60482	Pneumonia with Q-fever	pneumonia
72182	Pneumonia with salmonellosis	pneumonia
98782	Pneumonia with toxoplasmosis	pneumonia
49398	Pneumonia with typhoid fever	pneumonia
23726	Pneumonia with varicella	pneumonia
70559	Pneumonia with other infectious diseases EC NOS	pneumonia
66362	Pneumonia with infectious diseases EC NOS	pneumonia
886	Bronchopneumonia due to unspecified organism	pneumonia
16287	Chest infection - unspecified bronchopneumonia	pneumonia
572	Pneumonia due to unspecified organism	pneumonia
19400	Chest infection - pnemonia due to unspecified organism	pneumonia
9639	Lobar pneumonia due to unspecified organism	pneumonia
8318	Lung consolidation	pneumonia
3683	Basal pneumonia due to unspecified organism	pneumonia
34300	Postoperative pneumonia	pneumonia
15912	Influenza with pneumonia	pneumonia
29457	Chest infection - influenza with pneumonia	pneumonia
13573	Influenza with bronchopneumonia	pneumonia
62632	Influenza with pneumonia, influenza virus identified	pneumonia
35745	Influenza with pneumonia NOS	pneumonia
5324	Atypical pneumonia	pneumonia
35189	Abscess of lung with pneumonia	pneumonia
22835	Bronchiolitis obliterans organising pneumonia	pneumonia
52520	[X]Other viral pneumonia	pneumonia
63763	[X]Other bacterial pneumonia	pneumonia
98381	[X]Pneumonia due to other specified infectious organisms	pneumonia
53947	[X]Pneumonia in viral diseases classified elsewhere	pneumonia
53753	[X]Other pneumonia, organism unspecified	pneumonia
7577	gastric reflux	reflux
592	oesophagitis	reflux
2535	reflux oesophagitis	reflux
15054	acid reflux	reflux
7104	gastro-oesophageal reflux with oesophagitis	reflux
16605	oesophageal reflux with oesophagitis	reflux
15579	peptic oesophagitis	reflux
16450	regurgitant oesophagitis	reflux
4614	barrett's oesophagus	reflux
14760	oesophagitis nos	reflux
5596	barrett's ulcer of oesophagus	reflux
25610	oesopheal reflux without mention of oesophagitis	reflux
1327	oesophageal reflux	reflux
984	gastro-oesophageal reflux	reflux
2281	acid reflux	reflux
