# Supplementary material for: Health and cost impact of stepping down asthma medication for UK patients, 2001–2017: A population-based observational study
Source: PLoS Med. 2020 Jul 21;17(7):e1003145. doi: 10.1371/journal.pmed.1003145 (PMC7373267; doi:10.1371/journal.pmed.1003145)
Supplement: Attachment — Submitted filename: SteppingDown_ResponseToReviewers_5April2020.docx [file pmed.1003145.s012.docx]

**Response Letter: Health and cost impact of stepping-down asthma medication: a UK population-based observational study, 2001 - 2017**

Dear colleagues,

We would like to thank you for your comments, which have helped us to substantially improve the paper. In what follows, we provide a detailed response to each of the points you have raised.

**Requests from the editors:**

Please quote the study dates in the title.

These have been added.

In your abstract and throughout the paper, please add p values alongside 95% CI where available.

P values have now been included.

We ask you to quote aggregate demographic details for study participants in the abstract.

These have been added to the abstract.

Please add a new final sentence to the "Methods and findings" subsection of your abstract, quoting 2-3 of the study's main limitations.

Study limitations have now been added to the abstract.

At line 54, please begin the sentence with "In this study, we observed that ..." or similar.

This has been changed accordingly.

After your abstract, please add a new and accessible "author summary" section in non-identical prose. You may find it helpful to consult one or two recent research papers published in PLOS Medicine to get a sense of the preferred style.

Author summary is now added.

Early in the methods section of your main text, please state whether your study had a protocol or prespecified analysis plan (we note that you mention a protocol at line 204) please attach the document(s) as a supplementary file, referred to in the text. Please highlight analyses that were not prespecified.

We have added the protocol as a supplementary file.

Please restructure the early part of your discussion section so that the first paragraph provides a summary of the study's findings.

This has been changed as per your suggestion.

Please make that "include" at line 370.

This has been changed.

Throughout the paper, please quote exact p values or "p<0.001".

This has now been changed.

Throughout the text, please format reference call-outs as follows: "... people with asthma [1,2].".

This has now been changed.

In your reference list, please remove the academic editor's name from reference 4.

This has been removed.

Please attach a completed checklist for the most appropriate reporting guideline, which may be STROBE, as a supplementary document (referred to in your methods section). In the checklist, please refer to individual items by section (e.g., "Methods") and paragraph number rather than by line or page numbers, as the latter general change in the event of publication.

This has been added to the supplementary materials and referred to in the ethics and data statement.

**Comments from the reviewers:**

***** Reviewer #1:**

"Health and cost impact of stepping-down asthma medication: a UK population-based observational study" finds that asthma patients were bring prescribed higher levels of treatment oft without clear clinical indications, from nationwide UK primary care medical records from 2001 to 2017. Further analysis concluded that stepping-down medication to the minimum effective dose did not adversely affect outcomes, and moreover saved on costs. Indeed, stepping-down of ICS in particular consistently reduced hazard ratios (Tables S2/S3).

A major strength of this study would lie in the large number of patients involved (over 100,000 patients for each of the incident prescription/de-escalation analyses), from a cohort of over 500,000 patients from the UK primary care system over 17 years. Findings on the efficacy of stepping-down remained largely unchanged under relevant sensitivity analyses. To the best of our knowledge, there exists no prior study of comparable scale on the impacts of stepping-down asthma medication. Therefore, this study may have significant potential to affect health policy on asthma management, especially with an observed increase in the prescription of higher level medication, and purported insufficient evidence about stepping-down ICS medication as a factor in increased risk of future asthma attacks (e.g. from page 33 of the BTS/SIGN 2019 guidelines).

We thank you very much for your positive and encouraging feedback and the time taken to review our manuscript and make comments.

There however remain some points that might be clarified:

1. In lines 62-64, The appropriate citations might be provided for the various asthma guidelines (GINA, the Expert Panel Report, BTS/SIGN)

These have now been added.

2. From line 126, how was the criteria of >= 3 ICS prescriptions (parallel to infrequent ICS defined as <= 2 prescriptions per year in line 166) decided upon, given the mean of 7 prescriptions per year (line 133)? Data on the distribution of prescriptions/year might be illuminating.

An ICS cut-off was necessary to identify a study population of asthma patients that regularly use preventer medication, and therefore would be potentially suitable for stepping-down to a lower dose. We used our clinical judgement that 1-2 ICS per year suggested that the patient had very low requirement for ICS and would not require a reduction in their treatment dose. Furthermore, including such patients would bias the results towards unity if such a small dose was reduced. This has now been added to the methods section, page 9, line 172.

The distribution of 7 preventer prescriptions was only in the cohort included for stepping-down analysis (i.e. already had at least 3 preventer prescriptions). The interquartile range has been added to this to show the distribution, page 9, line 181.

3. On the definition of "stepping-down" (Line 138 onwards) - some possible configurations of medication step-down appear not to be explicitly covered by the chosen definition. For example, in prior work ("Stepping Down Asthma Treatment: How and When", Rogers & Reibman, Curr Opin Pulm Med. 2012 Jan; 18(1): 70-75.), it is claimed that "certain step-down practices such as the complete cessation of ICS to LABA alone was associated with a significant loss of asthma control". How might dropping ICS to an add-on therapy be categorized in the analysis (e.g. in Table 4 & 5)?

Patients that were stepped down to LABA mono therapy were not included as this is now a highly controversial management of asthma – current asthma guidelines, and FDA and MHRA, recommend that asthma patients should only be prescribed LABA in combination with ICS^1–3^.

4. More generally, how were the stepping-down definitions decided? Is halving dosage, for example, universally accepted practice as opposed to say a reduction of dosage by 25% (it is stated in line 309 that "The most common form of step down was halving ICS inhalations", but this does not seem to preclude other practices)?

The stepping-down definitions were based on BTS and GINA guidelines and the stepwise approach^1,2^. These guidelines suggest 25-50% can be used to step-down but most randomised controlled trials used 50% step-down and a larger step-down would be more likely to identify an effect if there was one^4^. This has been added to the methods, page 9, line 186.

5. The incidence rate (N) for the various drug-stepped-down categories might be provided in Tables 4 & 5, for convenience.

This has now been added to the legend below tables 4 as the effect estimate used was hazards/rates, but table 5 just N has been added, as the effect estimate used was odds during a one year follow-up period.

6. The definition of reliever prescriptions as a secondary outcome (e.g. line 154) might not be entirely obvious. The authors might consider adding a short description of preventer/reliever usage practices in the Introduction. Further, what is the trade off between reduced preventer and increased reliever usage?

Thank you for pointing this out, this has now been added, page 10, line 204. Increased reliever use with reduced preventer represents worse asthma control and usually inadequate preventer use.

7. On the incident analysis, it was stated that (line 229) "Of those prescribed higher level medication as their incident asthma prescription... 48.4% had not excerbation or reliever prescriptions in the previous year (Table 2)"; however, the 48.4% figure is not immediately evident from the presentation of Table 2.

Thank you, this has now been corrected.

8. Moreover, the authors might consider standardizing the formatting of Tables 2/S1/S4 (i.e. consistently place "N" and "%" as separate columns), and include p-values where appropriate.

Thank you for your comment, however, in all three tables ‘N’ is in the same column as the ‘%’. Only in Table S5 (was table S4 in the unrevised manuscript), showing a different analysis result, there is an additional column for total N. The analyses in Table 2 and S1 were performed to measure the proportions in each group having each comorbidity, therefore, as the analysis was not done to compare the number of people having comorbidities between the groups, no statistical analysis was carried out.

9. The characteristics examined for incident vs. step-down analysis (i.e. Table 2 vs. Table 3) are fairly different; for instance, age and socioeconomic status are not considered in the incident analysis, although it is not obvious that they do not affect/correlate with incident prescriptions. The authors might consider briefly discussing the selection of characteristics for each analysis.

The incident analysis was carried out to understand if the first inhaler was prescribed due to risk factors for worse asthma or presence of potential corticosteroid adverse effects, therefore age was included in the table, but not socioeconomic status. Socioeconomic status was included in the exacerbation model as a potential confounder as it has been found to be associated with exacerbations^5,6^. A discussion of selection of characteristics has now been added to the methods, page 8, line 164.

10. In line 236, it is stated that "(of) 125,341 patients, 39,897 were stepped-down, and 85,460 were not stepped down". However, the sum of stepped-down and non-stepped-down patients appears to be 125,357 patients.

Thank you for noticing this; it has now been corrected in the text.

11. Also, from the definition of stepping-down and the chronological length of the study (17 years), it seems possible that an individual (exposed) patient might step-down (and escalate) medication multiple times, over the full time period. How are such cases, if any, accounted for?

Only the first step-down that occurred with the eligibility criteria was used. Thank you for pointing this out, this has now been added to the methods, page 9, line 191. The main analysis was intention-to-treat and escalation was measured. A sensitivity analysis included as-treated approach (censoring patients on escalation if within 1-year). This was included in the methods in the statistical analysis, page 11, line 235.

12. From line 292, "Our study found most patients receiving a higher level preventer medication as their first asthma medication had not received a prescription for a preventer inhaler in the previous year (guidelines pre-2016 advised preventer therapy alone as the first treatment step(23))", is "preventer medication" not considered "preventer therapy"?

Thank you, this was a typo and now corrected to read ‘reliever therapy’.

13. In line 310, it is mentioned that step-down can be instigated by patients, possibly without direction from healthcare professionals. How does this happen in practice? Moreover, for the prescription data used in the analyses, were they taken as prescribed by the primary care health professionals (i.e. possibly not followed up on), or as filled by pharmacists?

Patients sometimes reduce the number of puffs they take on a daily basis without consulting a healthcare worker first - or if a healthcare worker has previously advised that as an option during annual asthma review or when given an asthma self-management plan. Therefore, they are not taking their inhalers as their prescription says. However, it is well known that most asthma patients do not fully adhere to their prescribed dose, and explains why very few patients have 9 or more preventer prescriptions per year^7–9^.

14. In line 355, which two studies do "both studies" refer to?

This was referring to the referenced study and our study but we can see this is not clear and have rectified that on page 21, line 417.

15. The discussion on prior investigations on medication step-down for asthma may be somewhat perfunctory. For example, it is claimed that (line 328) "Several randomised controlled trials of medication step-down have shown no difference in asthma exacerbations, asthma control, side-effects or quality of life"; however, citation (36) alone mentions that "Of 14 randomized controlled studies on step down of asthma medications (follow-up period range, 12-168 weeks), only 4 of them found no significant differences in asthma outcomes compared with patients maintaining their asthma medications".

Therefore, while there appears to be a general consensus from the latest asthma management guidelines on stepping-down controlled asthma cases to a minimal level, the evidence from previous randomized controlled trials appears somewhat mixed, even considering recognized methodological limitations. The authors might consider commenting in greater depth on these previous studies.

A fuller discussion of these trials has now been added, page 20, line 403 and is shown here for your convenience:

*‘A Cochrane Review, 2017, looked at studies that stepped-down ICS in well-controlled asthma patients (40). The review found six relevant studies; there was no difference in asthma exacerbations, asthma control, side-effects or quality of life. However, the quality of evidence was rated as low or very low. A Cochrane Review, 2015, looked at studies that stopped LABA in well-controlled adults. Five studies were reviewed, with moderate quality of evidence. However, trials were short with too few exacerbations to assess rates (41). A small study published since, found stopping LABA was well tolerated (42).’*

16. Finally, there may be some minor grammatical issues, e.g.

Line 39: "A cohort of patients were drawn..." -> "was drawn"

Line 47: "...and no increased in reliever" -> "no increase"

Line 297: "Perhaps not a surprising finding..." -> "This is perhaps not a surprising finding..."

Line 337: "this number was comparative..." -> "was comparable"?

Apologies. These have now all been corrected.

**References**

1.	British Thoracic Society. BTS/SIGN British guideline on the management of asthma. (2019).

2.	Global Strategy for Asthma Management and Prevention (2015 update).

3.	NHLBI. Expert panel report 3: guidelines for the diagnosis and management of asthma. *Natl. Hear. Lung, Blood Institute, Bethesda;* (2007).

4.	Crossingham, I., Evans, D. J., Halcovitch, N. R. & Marsden, P. A. Stepping down the dose of inhaled corticosteroids for adults with asthma. *Cochrane Database Syst. Rev.* (2017) doi:10.1002/14651858.CD011802.pub2.

5.	Bloom, C. I. *et al.* Exacerbation risk and characterisation of the UK’s asthma population from infants to old age. *Thorax* **73**, 313–320 (2018).

6.	Ramsahai, J. M., Hansbro, P. M. & Wark, P. A. B. Mechanisms and management of asthma exacerbations. *American Journal of Respiratory and Critical Care Medicine* vol. 199 423–432 (2019).

7.	McQuaid, E. L. Barriers to medication adherence in asthma: The importance of culture and context. *Annals of Allergy, Asthma and Immunology* vol. 121 37–42 (2018).

8.	McDonald, V. M. & Yorke, J. Adherence in severe asthma: time to get it right. *The European respiratory journal* vol. 50 (2017).

9.	Engelkes, M., Janssens, H. M., De Jongste, J. C., Sturkenboom, M. C. J. M. & Verhamme, K. M. C. Medication adherence and the risk of severe asthma exacerbations: A systematic review. *Eur. Respir. J.* **45**, 396–407 (2015).

***** Reviewer #2:**

This is an excellent study showing that asthma patients in the UK (1) are often initiated on overly intensive therapy, (2) are kept on intensive therapy without attempts to step down (contra guidelines), and (3) have no better outcomes than those whose therapy is stepped down.

Thank you very much for the time taken to review our manuscript and make comments, as well as your encouraging summary of our paper.

Major

1. My biggest concern is with the third finding—that stepping down therapy was not associated with more exacerbations or SABA use than continuation of therapy. As the authors recognize, there is a strong possibility for selection bias. They perform a stratified analysis based on exacerbations in the prior year. They also report in Tables 4 and 5 regression analyses that control for a number of potential confounders. These steps all help address the worry of unmeasured confounding. However, they did not perform the analysis with propensity score matching (or high-dimensional propensity score matching), which may help further allay concerns. In general, I worry that the group whose therapy was stepped down is different in important ways than the group whose therapy was continued.

Your point is well taken and we have therefore added this additional analysis to validate our results. Propensity score methods are indeed used to address a possible selection bias on observables when comparing two treatments administered to different samples. We constructed a PS using the same variables that were included in the Cox model. Inverse probability weighted PS were then used to estimate the average treatment time for the first exacerbation in stepped down patients, compared to those not stepped down. The result of this analysis has been added to the supplementary materials and shows negligible difference in days (Table S5) and page 15, line 307. The methods have been added to the methods section, page 12, line 243.

2. The authors did not report on any difference in the side-effects (diabetes, cataracts, glaucoma, osteopenia, or osteoporosis) in the group of patients who were stepped down compared to those who were not. Was there any improvement in these variables among those who were stepped down (whether fewer new diagnoses or improvement in symptoms [e.g. HgbA1c])?

We completely agree this is important and is a research question we are working on, but we believe it is unfortunately beyond the scope of this present study. To properly evaluate the effect of stepping down medication on side-effects requires a different study design, in part because the complex nature of the time-varying corticosteroid effect on the outcome.

3. This may be beyond the scope of data, but I wonder what proportion of patients had true asthma. As the authors allude to in the discussion (citing the paper by Aaron SD et al., JAMA 2017), many patients who carry a diagnosis of asthma and are started on inhalers never truly have asthma. What proportion of the patients in the cohort had PFTs or a methacholine challenge confirming a diagnosis of asthma? It is possible, as the authors acknowledge, that many patients did not have asthma—in which case, we would expect that stepping down medications would have no effect. Conversely, perhaps those with true asthma may have had more exacerbations and SABA use after being stepped down.

Unfortunately, this is not available from patients predominantly managed in primary care in the UK. Very few patients have PFTs or bronchoprovocation test from primary care and only around 4% of primary care asthma patients (Bloom et al, Journal of Asthma 2019) are seen in secondary care. Even those seen in secondary care, the results from such tests are infrequently recorded in the datasets used in our study, CPRD and HES. Interestingly, even those with a history of an exacerbation, therefore potentially more likely to truly have asthma, still did not exacerbate more after stepping-down. We have added this as a limitation to the study, page 23, line 467.

4. The authors note that, in the 107,908 patients in incident cohort, one-third were on an ICS and add-on, of which 94% were on a LABA. What proportion were on combination ICS-LABA versus two separate inhalers (ICS and LABA)? It would be interesting to examine whether being on a combination product affects the likelihood of being stepped down.

Thank you for this insightful comment. (a) The proportion on combination in the incident cohort was 77% for all incident inhalers between 2001 and 2017; by 2016 onwards 100% LABA-ICS were combination inhalers. This is now added, page14, line 284. (b) We have re-run the model including use of combination ICS-LABA as a variable. Combination inhalers were associated with a significantly reduced odds of having ICS stepped down, therefore including by reducing number of puffs, but interestingly did not significantly affect stepping-down LABA either way. The results (page 16, line 326), methods (page 11, line 216) and discussion (page 21, line 427) have been updated to report these additional findings; including clarifying this was a secondary analysis after your comment.

5. Of the 107,908 patients who were on an ICS-LABA combination, how many were on ICS-formoterol? The approach of using Symbicort as needed for mild asthma is new in the guidelines. But I wonder whether some clinicians in the UK were already doing this, in which case some of those on an ICS-LABA may not be getting stepped down because they are already on a low intensity therapy (i.e. ICS-formoterol PRN).

Thank you for raising this potential issue. However, in the UK during this period, Symbicort was only licensed to be used as SMART regimen (i.e. the simultaneous use of Symbicort as maintenance and reliever therapy in adults, allowing patients to increase the dose of Symbicort when their asthma worsens), not to be used only ‘as needed’. Furthermore, patients with very low use were not included in the study population for stepping-down analysis.

Minor

Line 77: This should be "is obtained with a low-dose" rather than "is obtained with low-dose."

Corrected

Line 110: This should be: "Prescription data were…" rather than "prescription data was..."

Corrected

Line 121: Does excluding non-HES linked patients affect the generalizability? Is there any reason to think that non-HES linked patients are different from HES-linked patients? This may be worth commenting on in the limitations.

Now added

Line 286: This should be "…a higher number of patients, around one-third, who were prescribed…" rather than "a higher number of patients, around one-third, that were prescribed…"

Corrected

Line 297: This should be "Perhaps this is not a surprising finding…" rather than "Perhaps not a surprising finding…"

Corrected

Line 373: This should be: "…excluding patients who decreased…" rather than "…excluding patients that decreased…"

Corrected

***** Reviewer #3:**

This is an analysis of the Clinical Practice Research Datalink (UK). The authors used observational data to describe asthma medication patterns with a focus on health outcomes and costs of stepping down asthma medications. The primary conclusion is that higher doses and numbers of asthma medications were prescribed in the UK from 2001-2017, and that most patients can safely reduce them, at a high cost savings.

We are very grateful for your time taken to review our manuscript and make comments.

Major comments:

1-One of the most interesting questions related to step down that was not considered in this analysis is listed as an asterisk in Table 1 (patients that stop all preventer meds were not included). Why not? I could not find in the methods section where this choice is explained. Stopping all controller meds (especially ICS) is probably the highest risk step down (see, for example, recent GINA guideline summarizing these risks). Also, it is potentially very relevant to new recommendations which suggest all asthma patients should use some level of ICS (intermittent or continuous). Without information on this step down risk, it is difficult to broadly state that step down moves do not adverse affect outcomes. I think the authors either need to analyze this group or be more precise in describing which step down types are likely low risk, and which were not analyzed.

Thank you for your comment. We did not include such patients as asthma guidelines refer to step-down as decreasing medication, but not completely stopping them. We also feel that patients in whom a non-specialist has decided to completely stop their medication, it is highly likely the physician does not feel they have asthma. Our study was solely intended to investigate the step-down/de-escalation of treatment. We agree that evaluating patients that complete stop is very important, but a different study that we hope to pursue in the future.

2-I think the following variables should be defined more clearly, as in how they were identified within the dataset and what they mean: "asthma management plan, annual asthma review, and inhaler technique check." I also wonder if having seen a specialist is another variable that should be included.

These variables have now been clarified (page 10, line 213), however, we were not able to include specialist review as a variable as we did not have the specific HES out-patient data for that variable for this cohort.

3-The final concluding statement (lines 391-394) which follows "In summary" focuses on not stepping up (which was a descriptive trend finding) and I think most of the analysis is related to outcomes of stepping down. Therefore, the authors may consider a summary statement that includes stepping down.

Thank you, a further concluding statement has now been added; page 24, line 487.

4-How to accomplish stepping down (i.e. how to solve this problem) is not clear from their data. Having annual visits, checking inhaler technique, and an asthma management plan do not seem to be related to step down. Are there any patterns from their data that suggest a solution? Are there signs of stepping down to ICS PRN and that this could be a successful strategy?

Our findings suggest that the most common form of stepping down is by reducing the number of puffs of ICS, which could include PRN, but this detail is unfortunately not available from the prescription data. It was beyond the scope of this quantitative study to understand why patients were not stepped down. As you note, from this data it appears it is not related to particular asthma specific clinical practices; this has now been commented on in the discussion, page 22, line 439 and here for your convenience:

*‘Notably, asthma specific clinical practices, including annual reviews and management plans, did not appear to increase the chances of stepping-down. A qualitative study of primary care health professionals suggested key factors why stepping-down does not occur are lack of confidence to step-down, lack of time to discuss and resistance from the patients (5).’*

Other comments:

1-Introduction section is concise and clear.

2-Sensitvity analyses are appropriate

3-Line 154-155--is there validation or a reference to justify 1 or more relievers as cut off?

The lowest cut-off possible was chosen as we deemed even a slight worsening of asthma control as reason not to step-down treatment but there has been no validation study for a particular SABA cut-off for change in asthma control.

4-Supplementary Figure 2 is very helpful. I suggest the authors consider moving this to the main document.

This has now been moved to the main figures.
